# Detection of Genetic Overlap Between Rheumatoid Arthritis and Systemic Lupus Erythematosus Using GWAS Summary Statistics

**DOI:** 10.3389/fgene.2021.656545

**Published:** 2021-03-18

**Authors:** Haojie Lu, Jinhui Zhang, Zhou Jiang, Meng Zhang, Ting Wang, Huashuo Zhao, Ping Zeng

**Affiliations:** ^1^Department of Epidemiology and Biostatistics, School of Public Health, Xuzhou Medical University, Xuzhou, China; ^2^Center for Medical Statistics and Data Analysis, School of Public Health, Xuzhou Medical University, Xuzhou, China

**Keywords:** rheumatoid arthritis, systemic lupus erythematosus, harmonic mean *P*-value, conjunction conditional false discover rate, pleiotropic genes

## Abstract

**Background:**

Clinical and epidemiological studies have suggested systemic lupus erythematosus (SLE) and rheumatoid arthritis (RA) are comorbidities and common genetic etiologies can partly explain such coexistence. However, shared genetic determinations underlying the two diseases remain largely unknown.

**Methods:**

Our analysis relied on summary statistics available from genome-wide association studies of SLE (*N* = 23,210) and RA (*N* = 58,284). We first evaluated the genetic correlation between RA and SLE through the linkage disequilibrium score regression (LDSC). Then, we performed a multiple-tissue eQTL (expression quantitative trait loci) weighted integrative analysis for each of the two diseases and aggregated association evidence across these tissues via the recently proposed harmonic mean *P*-value (HMP) combination strategy, which can produce a single well-calibrated *P*-value for correlated test statistics. Afterwards, we conducted the pleiotropy-informed association using conjunction conditional FDR (ccFDR) to identify potential pleiotropic genes associated with both RA and SLE.

**Results:**

We found there existed a significant positive genetic correlation (*r*_*g*_ = 0.404, *P* = 6.01E-10) via LDSC between RA and SLE. Based on the multiple-tissue eQTL weighted integrative analysis and the HMP combination across various tissues, we discovered 14 potential pleiotropic genes by ccFDR, among which four were likely newly novel genes (i.e., *INPP5B*, *OR5K2*, *RP11-2C24.5*, and *CTD-3105H18.4*). The SNP effect sizes of these pleiotropic genes were typically positively dependent, with an average correlation of 0.579. Functionally, these genes were implicated in multiple auto-immune relevant pathways such as inositol phosphate metabolic process, membrane and glucagon signaling pathway.

**Conclusion:**

This study reveals common genetic components between RA and SLE and provides candidate associated loci for understanding of molecular mechanism underlying the comorbidity of the two diseases.

## Introduction

Rheumatoid arthritis (RA) and systemic lupus erythematosus (SLE) are two frequent chronic rheumatic autoimmune diseases (Suresh, 2004; Cojocaru et al., 2011; Hu et al., 2019; Barbeira et al., 2020), with an incidence rate of 50 (Alamanos et al., 2006) or 8.6 (Stojan and Petri, 2018) per 100,000/year for RA or SLE in the United States, respectively, leading to growing disease burden worldwide among different ethnic, racial and age groups. Both RA and SLE have complicated etiologies, with genetic and hormonal factors (e.g., sex hormones, Tedeschi et al., 2013), environmental factors (e.g., cigarette smoking, Costenbader et al., 2004) and their interaction contributing to the onset and pathological processes of the two diseases (Salaman, 2003). Prior clinical and epidemiological studies have demonstrated that RA and SLE have overlapping clinical symptoms and increased familial aggregation (Alarcón-Segovia et al., 2005; Michou et al., 2008; Icen et al., 2009; Zhernakova et al., 2013; Acosta-Herrera et al., 2019; Ciccacci et al., 2019; James et al., 2019; Xiao et al., 2020), implying common susceptible mechanism and shared predisposition underlying two diseases.

One hypothesis to explain such comorbidity between RA and SLE is the common genetic etiology among autoimmune diseases (Cotsapas et al., 2011; Orozco et al., 2011; Suzuki et al., 2016; Márquez et al., 2017; Acosta-Herrera et al., 2019; Lehallier et al., 2019). Prior studies also provided evidence for shared genetic architecture of RA and SLE. For example, RA patients often have a higher incidence of HLA-DR4 genotype (chr6) compared to healthy controls (Stastny, 1978); meanwhile, correlation was well established between HLA class III (especially 6p21.3) and the susceptibility to develop SLE (Goldberg et al., 1976). Recently, genome-wide association studies (GWASs) have greatly advanced our knowledge of polygenic etiology of RA and SLE, and discovered a number of shared single nucleotide polymorphisms (SNPs) associated with them (Supplementary Table 1; Deng and Tsao, 2017; Okada et al., 2017). Examples of such shared loci include rs7574865 in *STAT4* (Remmers et al., 2007), rs2230926 and rs10499194 in *TNFAIP3* (Shimane et al., 2010), rs2476601 in *PTPN22* (Orozco et al., 2005), rs5754217 in *UBE2l3* (Orozco et al., 2011), rs9603612 nearby *COG6* (Márquez et al., 2017), as well as multiple loci in *NAB1*, *KPNA4*-*ARL14*, *DGQK*, *LIMK1*, and *PRR12* (Acosta-Herrera et al., 2019). Understanding these shared genetic determinants has significant implication for identifying important biomarkers and possesses the potential to develop novel therapeutic strategies for joint prediction, prevention, and intervention of RA and SLE.

However, the causal genes and pathways of RA and SLE remain largely unknown because, like many other diseases/traits (Manolio et al., 2009; Orozco et al., 2010), RA- or SLE-associated SNPs identified by GWASs explain only a very small fraction of phenotypic variance of RA or SLE (Julià Cano, 2011; Chen et al., 2017), suggesting that a large number of genetic variants with small to modest effect sizes (but still important) have yet not been discovered and that more pleiotropic genes would be discovered if increasing sample sizes (Wang et al., 2005; Tam et al., 2019). But increasing sample size is generally not feasible since recruiting and genotyping additional participants are expensive and time consuming. In addition, analyzing RA and SLE jointly with individual-level dataset is also difficult because of privacy concerns on data sharing (Pasaniuc and Price, 2016). Instead, summary statistics from large scale GWASs of RA and SLE are freely accessible (Okada et al., 2014; Bentham et al., 2015).

**FIGURE 1:**
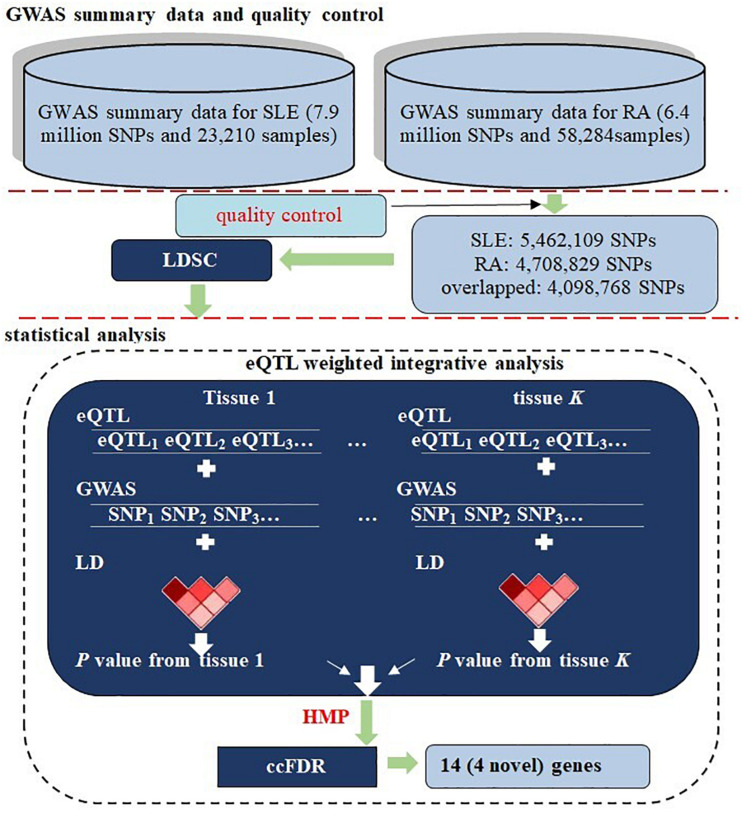
Flowchart of data preparation and analysis for RA and SLE. First, two sets of summary statistics were included for the two diseases; a series of quality control procedures were implemented and the genetic correlation between RA and SLE was evaluated using LDSC. Next, a multiple-tissue eQTL weighted integrative analysis was performed to aggregate association signals at the SNP level into the gene level, following by the HMP combination across tissues. Finally, cFDR was carried out to identify associated genes with pleiotropic effects. In the analysis, the LD was estimated with genotypes of the 1000 Genomes Project.

Therefore, a promising way is to apply genetic computational methods that efficiently analyze information contained in the existing pool of available GWAS summary statistics of RA and SLE for unveiling shared genetic contributors with pleiotropic effects more comprehensively. However, few of such studies on common genetic backgrounds of RA and SLE have been undertaken so far. To fill this literature gap, relying on summary statistics obtained from GWASs for SLE (*N* = 23,210) and RA (*N* = 58,284) (Okada et al., 2014; Bentham et al., 2015), in the present work we first evaluated the genetic correlation between the two diseases with cross-disease linkage disequilibrium score regression (LDSC) to quantify the extent of common genetic basis to which they share (Bulik-Sullivan et al., 2015). Next, we conducted an multiple-tissue eQTL (expression quantitative trait loci) weighted integrative analysis to aggregate the evidence of SNP-level associations into an integrative association at the gene level and then applied the recently proposed harmonic mean *P-*value (HMP) combination strategy (Wilson, 2019a) to combine a set of correlated *P*-values across various tissues into a single well-calibrated *P*-value. We further preformed the pleiotropy-informed association method using conditional false discovery rate (cFDR) (Andreassen et al., 2013; Smeland et al., 2020) to detect potentially pleiotropic genes. In total, we identified 14 genes that were associated with both RA and SLE, with four of them being likely newly novel pleiotropic genes. The flowchart of our data analysis is demonstrated in Figure 1.

## Materials and Methods

### GWAS Dataset for RA and SLE

We downloaded summary statistics (e.g., effect allele, effect size and *P*-value) of RA and SLE from public portal. Specifically, the GWAS of RA included 58,284 (14,361 cases and 43,923 controls) European individuals and 6,446,682 SNPs (Okada et al., 2014), while the GWAS of SLE included 23,210 (7,219 cases and 15,991 controls) individuals of European descent (Bentham et al., 2015) and 7,915,251 SNPs. Based on these summary statistics, we attempted to explore genetic overlap between RA and SLE at the gene level using novel statistical genetics approaches.

### Estimation of Cross-Disease Genetic Correlation

We first applied cross-disease LDSC to assess the genetic correlation *r*_*g*_ between RA and SLE (Bulik-Sullivan et al., 2015). The software of LDSC (version v1.0.1) was available at https://github.com/bulik/ldsc and implemented with default parameter settings. Following prior studies (Bulik-Sullivan et al., 2015), we performed stringent quality control before the LDSC analysis: (i) removed non-biallelic SNPs and those with strand-ambiguous alleles; (ii) excluded duplicated SNPs and those having no rs labels; (iii) excluded SNPs that were located within two special genetic regions including major histocompatibility complex (chr6: 28.5-33.3 Mb) (Bulik-Sullivan et al., 2015) and chr8: 7.2-12.5 Mb (Price et al., 2008) due to their complicated LD structure; (iv) kept SNPs that were included in the 1000 Genomes Project phase III (1000G); (v) removed SNPs whose allele did not match that in the 1000G.

The LD scores were computed using genotypes of 4,098,768 common SNPs (minor allele frequency > 0.01 and the *P*-value of Hardy Weinberg equilibrium test > 1E-5) with a 10 Mb window on 503 European individuals in the 1000G (Consortium, 2015); and then regressed them on the product of *Z* score statistics of RA and SLE. The regression slope of LDSC provides an unbiased estimate for *r*_*g*_ even when the samples are overlapped between the two GWASs of diseases (Bulik-Sullivan et al., 2015).

### Association Analysis by Integrating eQTL and GWAS Summary Statistics

Unlike prior studies which explored genetic overlap at the independent SNP level by using a pruning procedure (Lv et al., 2017; Peng et al., 2017; Hu et al., 2018a,b), we attempted to study common genetic component between RA and SLE at the gene level because gene is a more meaningful biological unit related to complex diseases compared with SNP. To do so, we performed the multiple-tissue eQTL weighted integrative analysis for a set of cis-SNPs located within a gene and produced a single *P*-value for the evidence of the significance of that gene (Gusev et al., 2016; Xu et al., 2017; Guo and Wu, 2018; Wu and Pan, 2018; Xue et al., 2020; Zhang et al., 2020). Specifically, for each tissue in turn and a set of predefined cis-SNPs of a gene of focus, we have:

(1) $$\chi_{k}^{2}={\left(\left({\text{Zw}}_{k}^{T}\right)/\sqrt{{\text{w}}_{k}\,{\text{Rw}}_{k}^{T}}\right)}^{2}$$

where **Z** = (*Z*_1_, …, *Z*_*m*_) is an *m*-vector of the *Z* score for cis-SNPs obtained from summary statistics, with *m* being the number of cis-SNPs and varying gene by gene across the whole genome; **w***_*k*_* is an *m*-vector of cis-SNP weights yielded from the *k*^*th*^ tissue of GTEx; **R** is the unknown LD among cis-SNPs and can be approximately estimated from reference panels such as the 1000G (Consortium, 2015). We performed our eQTL-weighted integrative analysis using the metaXcan software (Barbeira et al., 2018). For each gene its cis-SNPs were previously annotated by the authors of metaXcan and the eQTL weights were also in prior trained using the elastic net model with genotypes and gene expressions in tissues from the GTEx Project (GTEx Consortium, 2015). We downloaded tissue-specific eQTL weights from http://predictdb.org/ and run the integrative analysis in terms of the guideline of metaXcan (Barbeira et al., 2018).

Because both the RA and SLE GWASs were analyzed with pooled samples of male and female individuals; therefore, to avoid the influence of gender heterogeneity in gene expressions on association signals (Kassam et al., 2019; Lopes-Ramos et al., 2020; Oliva et al., 2020), we removed six gender-specific tissues in GTEx (i.e., breast mammary tissue, ovary, prostate, testis, uterus, and vagina), leaving 42 various gender-combined GTEx tissues for each disease (Supplementary Table 2). Moreover, due to population stratification or cryptic relatedness or overcorrection of test statistics (Cardon and Palmer, 2003; Freedman et al., 2004; van den Berg et al., 2019), the empirical null distribution in GWAS is sometimes inflated. To correct for such bias, following prior work (Price et al., 2010), we divided *$\chi_{k}^{2}$* by the genomic inflation factor (λ) if it was greater than 1.05. We estimated λ as the ratio between the observed median of *$\chi_{k}^{2}$* and the expected value of 0.456 (Devlin and Roeder, 1999; Dadd et al., 2009; Zeng et al., 2015b). Then, the *P-*value was easily calculated as the corrected test statistics asymptotically follow a chi-squared distribution with one degree of freedom (Gusev et al., 2016; Xu et al., 2017; Guo and Wu, 2018; Wu and Pan, 2018; Xue et al., 2020; Zhang et al., 2020). Afterwards, we obtained a set of various *P*-values (*P*_*k*_, *k* = 1, 2, …, 42) for every gene across these tissues, with each representing the association significance of the gene associated with RA or SLE after integrating eQTLs.

To aggregate individual association evidence across tissues, we further applied the HMP combination method to generate a single well-calibrated *P*-value (Wilson, 2019a):

(2) $$P=\int_{1/T}^{\infty }f_{x}\,\left(x|\log T+0.874,\frac{\pi }{2}\right)\,dx,T=1/\left(\sum_{k=1}^{K}\omega_{k}/P_{k}\right)$$

where *ω_*k*_* represents the non-negative weight for each *P*_*k*_ with _∑_k  =  1^Kω_k   =  1_ and assume that *ω_*k*_* is independent of *P*_*k*_; *f*_*x*_ denotes the Landau distribution probability density function. Note that, individual *P*_*k*_s often exhibited non-negligible positive dependence because they were implemented for the same gene following the similar logic (see below), existing methods such as the minimum *P*-value method (Conneely and Boehnke, 2007; Sun and Lin, 2020) and Fisher’s combination (Fisher, 1934), are either computationally intensive or rather difficult to implement because of the requirement of sampling-based algorithms for the valid null distribution or the unavailability of correlation structure (Ballard et al., 2010; Liu and Xie, 2019a,b; Zhang et al., 2019; Sun and Lin, 2020), especially when only summary-level datasets were available (Pasaniuc and Price, 2016). The advantage of HMP used here is that it has been theoretically demonstrated that the complicated positive dependency among *P-*values has little influence on the final pooled *P-*value (Wilson, 2019a). That is, *T* in the Eq. (2) still follows a Landau distribution asymptotically regardless of the correlation structure among these *P*-values. Consequently, we can yield the *P*-value for the test statistic *T* based on the right tail area of the Landau distribution as shown in (2). It has been also proven that under regularity conditions for the generalized central limit theorem the combined *P-*value by HMP is robust against the number of tests *K* and the selected weights (Wilson, 2019a). We implemented HMP with equal weights through the harmonic mean p package (version 3.0) in R (Wilson, 2019b). Using the HMP procedure, we generated two sets of *P*-values in the eQTL weighted gene-based association analyses of RA and SLE.

### Pleiotropy-Informed Method Identifying Shared Genes Between RA and SLE

Finally, to leverage the pleiotropic information shared between RA and SLE to efficiently identify gene association signals, we utilized the cFDR method (Andreassen et al., 2013, 2014; Smeland et al., 2020) which extended the unconditional FDR (Benjamini and Hochberg, 1995) from an empirical Bayes perspective. The cFDR measures the probability of the association of the principal disease (e.g., RA) conditioned on the strength of association with the conditional disease (e.g., SLE):

(3) $$\text{cFDR}\left(p_{i}|{\text{p}}_{j}\right)=Prob\left(H_{0}^{i}|p_{i}\leq p_{i},P_{j}\leq p_{j}\right)$$

where *p*_*i*_ and *p*_*j*_ are the observed HMP adjusted *P-*values of a particular gene of the principal disease (denoted by *i*) and the conditional disease (denoted by *j*), respectively; $H_{0}^{i}$ denotes the null hypothesis that there does not exist association between the gene and the principal disease. As the principal and conditional positions of the two diseases in cFDR are exchangeable, cFDR(*p*_*j*_| *p*_*i*_) is defined in a similar manner. Moreover, the conjunction conditional false discovery rate (ccFDR) is applied to identify genes with pleiotropic effect:

(4) $$\text{ccFDR}=\max\left\{\text{cFDR}\,\left(p_{i}|p_{j}\right),\text{cFDR}\,\left(p_{j}|p_{i}\right)\right\}$$

which is defined as the probability that a given gene has a false positive association with both the principal and conditional diseases, and provides an indicator for pleiotropy (Andreassen et al., 2013; Smeland et al., 2020).

## Results

### Estimated Genetic Correlation Between RA and SLE

After quality control, a total of 4,708,829 and 5,462,109 genetic variants were reserved for RA and SLE, respectively. The genome-wide SNP-based heritability is estimated to be 11.8% (*se* = 1.3%) for RA and 30.1% (*se* = 3.1%) for SLE with LDSC. Next, using cross-disease LDSC with 4,098,768 common SNPs, we observe that there exists a significantly positive genetic correlation between the two types of diseases (*r_*g*_* = 0.404, *P* = 6.01E-10), providing statistical evidence supporting overlapped genetic foundation between RA and SLE. Overall, through the above genetic correlation analysis we reveal RA and SLE are genetically similar and share moderate to high overlap in genetic etiology. Therefore, it is worthy of further investigation into common genetic mechanisms through novel pleiotropy-informed statistical tools.

### Associated Genes Identified by cFDR

**FIGURE 2:**
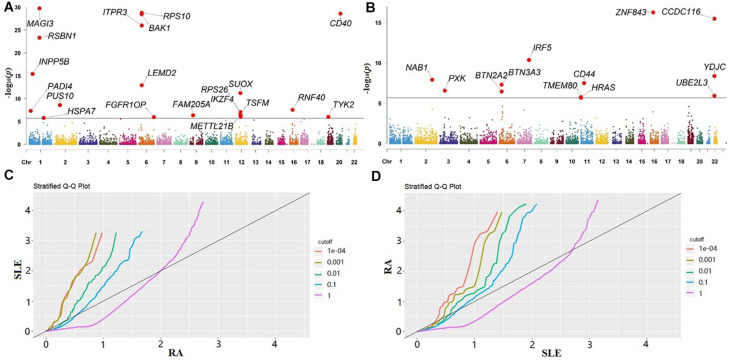
**(A)** Manhattan plot for RA; **(B)** Manhattan plot for SLE. The genes in both plots with *P*-value < 2.1E-06 (the significance level adjusted by Bonferroni’s method) are highlighted; **(C)** Q-Q plot of RA conditional on the nominal *P-*value of SLE; **(D)** Q-Q plot of SLE conditional on the nominal *P-*value of RA. RA, rheumatoid arthritis; SLE, systemic lupus erythematosus.

In our gene-based association analysis, we assigned a set of genetic variants to predefined genes and obtained a total of 23,833 and 23,813 genes for RA or SLE, respectively. By integrating eQTLs and summary statistics, we generated *$\chi_{k}^{2}$* for each gene of both RA and SLE across all the tissues and adjusted it if λ > 1.05 (Supplementary Table 3). Then, the *P*-values were yielded. Afterwards, we further performed the HMP procedure to aggregate the *P*-values of each gene across all the tissues into a single *P*-value for RA or SLE. As mentioned before, these *P*-values from various tissues are in highly positive correlation with each other (Supplementary Figure 1), implying the failure if applying Fisher’s method which instead requires mutually independent *P*-values from different experiment tests (Fisher, 1934; Rice, 2010). The Manhattan plots for RA and SLE are shown in Figures 2A,B, with some associated genes highlighted.

According to the results of HMP, we conducted the cFDR analysis. In our analysis the Q-Q plot of RA conditional on the nominal *P-*value of SLE illustrates the existence of enrichment at different significance thresholds of SLE (Figure 2C). The presence of leftward shift suggests that the proportion of true associations for a given *P*-value of SLE would increase when the analysis is limited to include more significant genes. On the other hand, in terms of the Q-Q plot of SLE conditional on the nominal *P*-value of RA (Figure 2D), we observe a more pronounced separation in different curves, implying that there exists a stronger enrichment for SLE given RA than that for RA given SLE.

We further formally analyze the two diseases jointly using cFDR and show the results of association signals in Table 1 and Supplementary Tables 4, 5. Briefly, we identify 76 RA-associated genes (Supplementary Table 4) and 33 SLE-associated genes (cFDR < 0.05) (Supplementary Table 5). Among these genes, 59 RA-associated (e.g., *CCBL2*, *SLC10A4*, and *PLEKHA1*) and 19 SLE-associated genes (e.g., *INPP5B*, *SKP1*, and *TMEM80*) are not implied in the original GWASs of RA and SLE (Okada et al., 2014; Bentham et al., 2015), and are likely newly candidate associated genes for each disease. These findings also confirm that our multiple-tissue eQTL weighted integrative gene-based association analysis has higher power compared to the conventional single SNP analysis, as shown in many prior studies (Gusev et al., 2016; Xu et al., 2017; Guo and Wu, 2018; Wu and Pan, 2018; Xue et al., 2020; Zhang et al., 2020).

**TABLE 1:** Potential pleiotropic genes associated with RA and SLE identified by ccFDR.

**Gene**	**chr**	**Position**	**cFDR_*RA*_**	**cFDR_*SLE*_**	**ccFDR**
*INPP5B*	1	38,326,369-38,412,729	3.91E-16	5.64E-3	2.54E-2
*OR5K2*	3	98,216,448-98,217,496	1.42E-2	9.12E-4	4.63E-2
*RP11-2C24.5*	16	30,832,389-30,833,431	1.86E-2	2.34E-16	3.72E-2
*CTD-3105H18.4*	19	12,490,560-12,494,501	1.37E-2	2.79E-4	4.45E-2
*MAGI3*	1	113,933,371-114,228,545	1.81E-30	1.60E-2	1.60E-2
*INPP1*	2	191,208,196-191,236,391	7.07E-3	5.79E-5	4.24E-2
*PDHB*	3	58,413,357-58,419,584	2.51E-5	5.66E-4	6.60E-3
*ITPR3*	6	33,588,522-33,664,351	9.83E-27	4.60E-4	2.76E-3
*CLIP2*	7	73,703,803-73,820,273	4.88E-3	7.32E-5	3.09E-2
*IRF5*	7	128,577,666-128,590,089	5.45E-4	4.27E-11	2.72E-3
*ICAM5*	19	10,400,655-10,407,453	1.90E-3	1.57E-4	1.09E-2
*TYK2*	19	10,461,209-10,491,352	1.01E-6	2.57E-5	5.39E-4
*CCDC116*	22	21,987,007-21,991,616	1.06E-2	3.38E-16	3.18E-2
*RP11-387H17.4*	17	39,927,742-39,939,601	4.14E-5	1.06E-4	2.17E-3

*The first four may be newly pleiotropic genes, while the others were reported in previous literature.*

### Pleiotropic Genes Identified by ccFDR

Across all these RA- and SLE-associated genes, 14 genes (i.e., pleiotropic genes) are commonly related to RA and SLE (ccFDR < 0.05) (Table 1), four of which (i.e., *INPP5B*, *OR5K2*, *RP11-2C24.5*, and *CTD-3105H18.4*) are possibly new genes. Interestingly, the SNP effect sizes of 12 out of 14 genes (except *PDHB* and *RP11-387H17.4*) are highly positively correlated between RA and SLE (Supplementary Figure 2), with an average Pearson’s correlation *r* of 0.579. For example, the SNP effect sizes of *IRF5* have a maximal *r* of 0.862 (95% confidence intervals [CIs] 0.826-0.891), followed by *INPP1* (*r* = 0.825, 95%CIs 0.792-0.853) and *TYK2* (*r* = 0.755, 95%CIs 0.701-0.799). This observation indicates that the genetic effects of these pleiotropic genes on the two diseases in general show a consistent direction. In addition, we also calculated the genetic risk score (GRS) for each pleiotropic gene (Supplementary Figure 3). The GRS is generated as the product of SNP effect sizes of RA (or SLE) and genotypes available from 503 European individuals of the 1000G (Consortium, 2015). We applied the GRS as an overall measurement of the genetic effect for a given pleiotropic gene on each disease. We observe that most of these pleiotropic genes (except *INPP5B* and *RP11-2C24.5*) have substantial different average GRS on RA and SLE. For example, five (i.e., *OR5K2*, *INPP1*, *PDHB*, *ITPR3*, and *ICAM5*) show a higher overall genetic effect on SLE, while the rest (i.e., *CTD-3105H18.4*, *MAG13*, *CLIP2*, *INF5*, *TYK2*, *CCDC116*, and *RP11-387H17.4*) show a higher overall genetic effect on RA. These GRS results provide an insight into the magnitude of the genetic effects of these pleiotropic genes on the two diseases.

The result of enrichment analysis shows that these pleiotropic genes have marked enrichment patterns mainly in type I interferon (IFN) signaling pathway, membrane, and inositol phosphate metabolic process (Table 2). The IFN signaling pathway plays a major role in activation of both innate and adaptive immune systems that are related to RA (Wright et al., 2015) and SLE (Bezalel et al., 2014). *IRF5* and *TYK2* are shown to be involved in this pathway, in line with prior studies (Acosta-Herrera et al., 2019) and supporting the validity of our results. Some pleiotropic genes (e.g., *INPP5B*, *MAGI3*, *ITPR3*, *TYK2*, and *ICAM5*) are enriched in the biological process of membrane fraction. *MAGI3*, as a novel membrane-associated guanylate kinase, is also implicated in the Wnt/β-catenin pathway, which induces promotion of regulatory T cell responses (Norén et al., 2017) as well as immune tolerance and plays a critical role in mucosal tolerance and suppression of chronic autoimmune pathologies (Suryawanshi et al., 2016). *INPP5B*, enriched in the pathway of inositol phosphate metabolic process and membrane fraction, was recently reported to be associated with membranes through an isoprenyl modification near the C-terminus and regulated calcium signaling by inactivating inositol phosphates (Nakatsu et al., 2015). Sustained calcium signaling responses are prevalent in the immunological synapse of T cells of SLE patients (Nicolaou et al., 2010). In addition, the mobilization of calcium signaling may modulate the functions of inflammatory and immunity genes in RA patients (de Seabra Rodrigues Dias et al., 2017).

**TABLE 2:** Enriched pathways for potential pleiotropic genes of RA and SLE.

**Pathway**	**Genes**	***P***
Phosphatidylinositol signaling system	*INPP5B*, *INPP1*, *ITPR3*	4.00E-3
Inositol phosphate metabolic process	*INPP5B*, *INPP1*	2.20E-2
Membrane	*INPP5B*, *MAGI3*, *ITPR3*, *TYK2*, *ICAM5*	2.40E-2
Type I interferon signaling pathway	*IRF5*, *TYK2*	3.00E-2
Inositol phosphate metabolism	*INPP5B*, *INPP1*	7.00E-2
Glucagon signaling pathway	*ITPR3*, *PDHB*	9.70E-2

As another example, *PDHB*, enriched in the glucagon signaling pathway, was discovered to be related to the promotion of aerobic glucose metabolism together with oxidative stress (Kanda et al., 2015). Up-regulation of glucose metabolism was demonstrated to be associated with upon activation of immune cells such as Fibroblast-like Synoviocytes (FLSs) (Garcia-Carbonell et al., 2016). The involvement of FLSs in regulating the pathogenesis of RA was highlighted in recent work (Meng and Qiu, 2020), which supports the validity of our findings.

## Discussion

It has been widely observed that RA and SLE have common pathological and clinical features (Manoussakis et al., 2004; Icen et al., 2009; Orozco et al., 2011; Toro-Domínguez et al., 2014; Márquez et al., 2017; Acosta-Herrera et al., 2019), which are partly attributable to common genetic foundation between the two diseases. However, the genetic overlap underlying RA and SLE remains elusive and a large proportion of genes related to them are yet not discovered (Ramos et al., 2011). Large-scale GWASs undertaken on RA and SLE offer an unprecedented opportunity to tackle this question. The objective of our study was to gain insight into genetic mechanism linking between RA and SLE using advanced bioinformatics approaches. By leveraging publicly available GWAS summary statistics, we identified a significant positive genetic correlation between RA and SLE, indicating that genetic variants associated with the risk of SLE would be also related to the risk of RA, or vice versa (Icen et al., 2009; Toro-Domínguez et al., 2014).

By integrating eQTLs and combing association evidence across tissues, our study ultimately discovered 14 potential pleiotropy genes, and four of them (i.e., *INPP5B*, *OR5K2*, *RP11-2C24.5* and *CTD-3105H18.4*) were not directly reported in previous literature, providing new insight into shared genetic basis between RA and SLE. Furthermore, we found that the SNP effect sizes of these genes were positively correlated and that these genes were implicated in multiple auto-immune relevant pathways such as inositol phosphate metabolic process, membrane and glucagon signaling pathway. As compared to previous cross-phenotype studies of autoimmune diseases (Orozco et al., 2011; Márquez et al., 2017), our study differs from them in multiple aspects and has several strengths. First, previous studies in general attempted to detect pleiotropy loci at the SNP level, while our work aimed to identify shared loci with gene as the functional unit. The power for detecting single SNP association is limited because genetic variants often have weak effect sizes (Hindorff et al., 2009; Visscher et al., 2017), making the detection of common associated SNPs difficult even with large samples. In contrast, due to the aggregation of multiple weak association signals and the reduced burden of multiple testing, gene-based analysis often has higher power than its counterpart of single SNP analysis. Therefore, our analysis is biologically more meaningful and statistically more powerful as widely demonstrated by gene-based association studies (Kwee et al., 2008; Wu et al., 2010, 2011; Schifano et al., 2012; Huang and Lin, 2013; Ionita-Laza et al., 2013; Wang et al., 2013; Lee et al., 2014; Zeng et al., 2014a,b, 2015a).

Second, as shown in previous studies, disease-associated SNPs were more likely to be eQTLs (Nicolae et al., 2010), implying that the functional roles of associated SNPs were regulated through gene expression; thus, the power improvement of gene-based association studies would be further achieved by integrating eQTLs into the test (Su et al., 2018; Wu and Pan, 2018; Xue et al., 2020). To do so, we systematically evaluate predicted gene expressions in RA and SLE through integrating eQTLs of relevant tissues from the GTEx project and GWAS summary statistics. Third, we aggregated association evidence across various tissues by applying the HMP procedure (Wilson, 2019a) which is robust against positive dependency among *P*-values and can produce a single well-calibrated *P*-value for evaluating the association. Fourth, prior studies were performed as across-disease meta-analysis for RA and SLE aimed to detected genetic loci that were associated with at least one disease rather than simultaneously related to both the diseases (Orozco et al., 2011; Márquez et al., 2017; Acosta-Herrera et al., 2019). Compared to these studies, besides the HMP combination strategy, our work also applied the widely used pleiotropy-informed method of cFDR to formally discover shared genes and provided a solid statistical foundation for our analysis (Zhernakova et al., 2009; Andreassen et al., 2013, 2014; Li et al., 2015; Ellinghaus et al., 2016; Smeland et al., 2020).

Finally, there are some limitations of our study needed to state. First, we cannot replicate these identified pleiotropic genes with external datasets or via in vivo and in vitro experiments. Second, the limited sample size of reference panel for calculating LD among SNPs and the well-known tissue-specific genetic effects of eQTLs may undermine the power of our integrative analysis. Third, although prior evidence described before indicates these newly pleiotropic genes may underlie certain aspects of the pathogenesis of RA and SLE, their biological mechanisms are still largely unclear; therefore, further studies are required to characterize functional roles of these genes on RA and SLE.

## Conclusion

This study reveals common genetic components between RA and SLE and provides candidate associated loci for understanding of molecular mechanism underlying the comorbidity of the two diseases.

## Data Availability Statement

The original contributions presented in the study are included in the article/Supplementary Material, further inquiries can be directed to the corresponding author/s.

## Author Contributions

PZ conceived the idea for the study. PZ and HL interpreted the results of the data analyses. PZ, JZ, and HL wrote the manuscript with help from other authors. All authors obtained the data and performed the data analyses.

## Conflict of Interest

The authors declare that the research was conducted in the absence of any commercial or financial relationships that could be construed as a potential conflict of interest. The handling editor declared a past co-authorship with one of the authors PZ

## Abbreviations

1000G: 1000 genomes project phase III
95% CIs: 95% confidence intervals
ccFDR: conjunction conditional FDR
eQTL: expression quantitative trait locus
FLSs: fibroblast-like synoviocytes
GTEx: genotype-tissue expression
GWAS: genome-wide association study
HMP: harmonic mean *P*-value
LDSC: linkage disequilibrium score regression
RA: rheumatoid arthritis
SLE: systemic lupus erythematosus.

## References

[B1] Acosta-Herrera M. Kerick M. González-Serna D. Wijmenga C. Franke A. Gregersen P. K. (2019). Genome-wide meta-analysis reveals shared new loci in systemic seropositive rheumatic diseases. *Ann. Rheum Dis.* 78 311–319. 10.1136/annrheumdis-2018-214127 30573655 PMC6800208

[B2] Alamanos Y. Voulgari P. V. Drosos A. A. (2006). Incidence and prevalence of rheumatoid arthritis, based on the 1987 American College of Rheumatology criteria: a systematic review. *Semin. Arthr. Rheumat.* 36 182–188. 10.1016/j.semarthrit.2006.08.006 17045630

[B3] Alarcón-Segovia D. Alarcón-Riquelme M. E. Cardiel M. H. Caeiro F. Massardo L. Villa A. R. (2005). Familial aggregation of systemic lupus erythematosus, rheumatoid arthritis, and other autoimmune diseases in 1,177 lupus patients from the GLADEL cohort. *Arthr. Rheumat.* 52 1138–1147. 15818688 10.1002/art.20999

[B4] Andreassen O. A. Djurovic S. Thompson W. K. Schork A. J. Kendler K. S. O’Donovan M. C. (2013). Improved detection of common variants associated with schizophrenia by leveraging pleiotropy with cardiovascular-disease risk factors. *Am. J. Hum. Genet.* 92 197–209. 23375658 10.1016/j.ajhg.2013.01.001 PMC3567279

[B5] Andreassen O. A. Zuber V. Thompson W. K. Schork A. J. Bettella F. Practical Consortium (2014). Shared common variants in prostate cancer and blood lipids. *Int. J. Epidemiol.* 43 1205–1214. 10.1093/ije/dyu090 24786909 PMC4121563

[B6] Ballard D. H. Cho J. Zhao H. (2010). Comparisons of multi-marker association methods to detect association between a candidate region and disease. *Genet. Epidemiol.* 34 201–212. 19810024 10.1002/gepi.20448 PMC3158797

[B7] Barbeira A. N. Dickinson S. P. Bonazzola R. Zheng J. Wheeler H. E. Torres J. M. (2018). Exploring the phenotypic consequences of tissue specific gene expression variation inferred from GWAS summary statistics. *Nat. Commun.* 9:1825. 10.1038/s41467-018-03621-1 29739930 PMC5940825

[B8] Barbeira A. N. Melia O. J. Liang Y. Bonazzola R. Wang G. Wheeler H. E. (2020). Fine−mapping and QTL tissue−sharing information improves the reliability of causal gene identification. *Genet. Epidemiol.* 44 854–867. 10.1002/gepi.22346 PMC7693040 32964524

[B9] Benjamini Y. Hochberg Y. (1995). Controlling the false discovery rate: a practical and powerful approach to multiple testing. *J. R. Statist. Soc. Ser. B (Statist. Methodol.)* 57 289–300.

[B10] Bentham J. Morris D. L. Graham D. S. C. Pinder C. L. Tombleson P. Behrens T. W. (2015). Genetic association analyses implicate aberrant regulation of innate and adaptive immunity genes in the pathogenesis of systemic lupus erythematosus. *Nat. Genet.* 47 1457–1464. 10.1038/ng.3434 26502338 PMC4668589

[B11] Bezalel S. Guri K. M. Elbirt D. Asher I. Sthoeger Z. M. (2014). Type I interferon signature in systemic lupus erythematosus. *Isr. Med. Assoc. J.* 16 246–249. 24834763

[B12] Bulik-Sullivan B. Finucane H. K. Anttila V. Gusev A. Day F. R. Loh P.-R. (2015). An atlas of genetic correlations across human diseases and traits. *Nat. Genet.* 47:1236. 10.1038/ng.3406 PMC4797329 26414676

[B13] Cardon L. R. Palmer L. J. (2003). Population stratification and spurious allelic association. *Lancet* 361 598–604. 12598158 10.1016/S0140-6736(03)12520-2

[B14] Chen L. Morris D. L. Vyse T. J. (2017). Genetic advances in systemic lupus erythematosus: an update. *Curr. Opin. Rheumatol.* 29 423–433. 28509669 10.1097/BOR.0000000000000411

[B15] Ciccacci C. Latini A. Perricone C. Conigliaro P. Colafrancesco S. Ceccarelli F. (2019). TNFAIP3 gene polymorphisms in three common autoimmune diseases: systemic lupus erythematosus, rheumatoid arthritis, and primary sjogren syndrome—association with disease susceptibility and clinical phenotypes in italian patients. *J. Immunol. Res.* 2019:6728694. 10.1155/2019/6728694 31534975 PMC6732636

[B16] Cojocaru M. Cojocaru I. M. Silosi I. Vrabie C. D. (2011). Manifestations of systemic lupus erythematosus. *Maedica (Buchar)* 6 330–336. PMC3391953 22879850

[B17] Conneely K. N. Boehnke M. (2007). So many correlated tests, so little time! Rapid adjustment of P values for multiple correlated tests. *Am. J. Hum. Genet.* 81 1158–1168. 17966093 10.1086/522036 PMC2276357

[B18] Consortium G. P. (2015). A global reference for human genetic variation. *Nature* 526 68–74. 26432245 10.1038/nature15393 PMC4750478

[B19] Costenbader K. H. Kim D. J. Peerzada J. Lockman S. Nobles−Knight D. Petri M. (2004). Cigarette smoking and the risk of systemic lupus erythematosus: a meta−analysis. *Arthr. Rheumat.* 50 849–857. 15022327 10.1002/art.20049

[B20] Cotsapas C. Voight B. F. Rossin E. Lage K. Neale B. M. Wallace C. (2011). Pervasive sharing of genetic effects in autoimmune disease. *PLoS Genet.* 7:e1002254. 10.1371/journal.pgen.1002254 21852963 PMC3154137

[B21] Dadd T. Weale M. E. Lewis C. M. (2009). A critical evaluation of genomic control methods for genetic association studies. *Genet. Epidemiol.* 33 290–298. 10.1002/gepi.20379 19051284

[B22] de Seabra Rodrigues Dias I. R. Mok S. W. F. Gordillo-Martínez F. Khan I. Hsiao W. W. L. Law B. Y. K. (2017). The calcium-induced regulation in the molecular and transcriptional circuitry of human inflammatory response and autoimmunity. *Front. Pharmacol.* 8:962. 10.3389/fphar.2017.00962 29358919 PMC5766673

[B23] Deng Y. Tsao B. P. (2017). Updates in lupus genetics. *Curr. Rheumatol. Rep.* 19:68. 10.1007/s11926-017-0695-z 28983873

[B24] Devlin B. Roeder K. (1999). Genomic control for association studies. *Biometrics* 55 997–1004. 11315092 10.1111/j.0006-341x.1999.00997.x

[B25] Ellinghaus D. Jostins L. Spain S. L. Cortes A. Bethune J. Han B. (2016). Analysis of five chronic inflammatory diseases identifies 27 new associations and highlights disease-specific patterns at shared loci. *Nat. Genet.* 48 510–518. 26974007 10.1038/ng.3528 PMC4848113

[B26] Fisher R. A. (1934). “Statistical methods for research workers,” in *Biological Monographs and Manuals*, 5 Edn, eds Kotz S. Johnson N. L. (Edinburgh: Oliver and Boyd Ltd).

[B27] Freedman M. L. Reich D. Penney K. L. McDonald G. J. Mignault A. A. Patterson N. (2004). Assessing the impact of population stratification on genetic association studies. *Nat. Genet.* 36 388–393. 10.1038/ng1333 15052270

[B28] Garcia-Carbonell R. Divakaruni A. S. Lodi A. Vicente-Suarez I. Saha A. Cheroutre H. (2016). Critical role of glucose metabolism in rheumatoid arthritis fibroblast-like synoviocytes. *Arthr. Rheumatol.* 68 1614–1626. 10.1002/art.39608 26815411 PMC4963240

[B29] Goldberg M. A. Arnett F. C. Bias W. B. Shulman L. E. (1976). Histocompatibility antigens in systemic lupus erythematosus. *Arthr. Rheumat. Offic. J. Am. Coll. Rheumatol.* 19 129–132. 10.1002/art.1780190201 1259797

[B30] GTEx Consortium (2015). The Genotype-Tissue Expression (GTEx) pilot analysis: multitissue gene regulation in humans. *Science* 348 648–660. 10.1126/science.1262110 25954001 PMC4547484

[B31] Guo B. Wu B. (2018). Statistical methods to detect novel genetic variants using publicly available GWAS summary data. *Comput. Biol. Chem.* 74 76–79. 10.1016/j.compbiolchem.2018.02.016 29558699 PMC6159229

[B32] Gusev A. Ko A. Shi H. Bhatia G. Chung W. Penninx B. W. (2016). Integrative approaches for large-scale transcriptome-wide association studies. *Nat. Genet.* 48 245–252. 26854917 10.1038/ng.3506 PMC4767558

[B33] Hindorff L. A. Sethupathy P. Junkins H. A. Ramos E. M. Mehta J. P. Collins F. S. (2009). Potential etiologic and functional implications of genome-wide association loci for human diseases and traits. *Proc. Natl. Acad. Sci. U.S.A.* 106 9362–9367. 10.1073/pnas.0903103106 19474294 PMC2687147

[B34] Hu Y. Li M. Lu Q. Weng H. Wang J. Zekavat S. M. (2019). A statistical framework for cross-tissue transcriptome-wide association analysis. *Nat. Genet.* 51 568–576. 10.1038/s41588-019-0345-7 30804563 PMC6788740

[B35] Hu Y. Tan L.-J. Chen X.-D. Greenbaum J. Deng H.-W. (2018a). Identification of novel variants associated with osteoporosis, type 2 diabetes and potentially pleiotropic loci using pleiotropic cFDR method. *Bone* 117 6–14. 10.1016/j.bone.2018.08.020 30172742 PMC6364698

[B36] Hu Y. Tan L.-J. Chen X.-D. Liu Z. Min S.-S. Zeng Q. (2018b). Identification of novel potentially pleiotropic variants associated with osteoporosis and obesity using the cFDR method. *J. Clin. Endocrinol. Metab.* 103 125–138. 10.1210/jc.2017-01531 29145611 PMC6061219

[B37] Huang Y. T. Lin X. (2013). Gene set analysis using variance component tests. *BMC Bioinform.* 14:210. 10.1186/1471-2105-14-210 PMC3776447 23806107

[B38] Icen M. Nicola P. J. Maradit-Kremers H. Crowson C. S. Therneau T. M. Matteson E. L. (2009). Systemic lupus erythematosus features in rheumatoid arthritis and their effect on overall mortality. *J. Rheumatol.* 36 50–57. 10.3899/jrheum.080091 19004043 PMC2836232

[B39] Ionita-Laza I. Lee S. Makarov V. Buxbaum, Joseph D. Lin X. (2013). Sequence kernel association tests for the combined effect of rare and common variants. *Am. J. Hum. Genet.* 92 841–853. 10.1016/j.ajhg.2013.04.015 23684009 PMC3675243

[B40] James J. A. Chen H. Young K. A. Bemis E. A. Seifert J. Bourn R. L. (2019). Latent autoimmunity across disease-specific boundaries in at-risk first-degree relatives of SLE and RA patients. *EBioMedicine* 42 76–85. 30952617 10.1016/j.ebiom.2019.03.063 PMC6491794

[B41] Julià Cano A. (2011). *Genomic Approaches for the Identi Cation of Risk Loci for Rheumatoid Arthritis.* Bellaterra: Universitat Autònoma de Barcelona.

[B42] Kanda A. Noda K. Ishida S. (2015). ATP6AP2/(pro) renin receptor contributes to glucose metabolism via stabilizing the pyruvate dehydrogenase E1 β subunit. *J. Biol. Chem.* 290 9690–9700. 25720494 10.1074/jbc.M114.626713 PMC4392269

[B43] Kassam I. Wu Y. Yang J. Visscher P. M. McRae A. F. (2019). Tissue-specific sex-differences in human gene expression. *Hum. Mol. Genet.* 28 2976–2986. 10.1093/hmg/ddz090 31044242 PMC6736104

[B44] Kwee L. C. Liu D. Lin X. Ghosh D. Epstein M. P. (2008). A powerful and flexible multilocus association test for quantitative traits. *Am. J. Hum. Genet.* 82 386–397. 10.1016/j.ajhg.2007.10.010 18252219 PMC2664991

[B45] Lee S. Abecasis, Gonçalo R. Boehnke M. Lin X. (2014). Rare-variant association analysis: study designs and statistical tests. *Am. J. Hum. Genet.* 95 5–23. 10.1016/j.ajhg.2014.06.009 24995866 PMC4085641

[B46] Lehallier B. Gate D. Schaum N. Nanasi T. Lee S. E. Yousef H. (2019). Undulating changes in human plasma proteome profiles across the lifespan. *Nat. Med.* 25 1843–1850. 10.1038/s41591-019-0673-2 31806903 PMC7062043

[B47] Li Y. R. Li J. Zhao S. D. Bradfield J. P. Mentch F. D. Maggadottir S. M. (2015). Meta-analysis of shared genetic architecture across ten pediatric autoimmune diseases. *Nat. Med.* 21 1018–1027. 10.1038/nm.3933 26301688 PMC4863040

[B48] Liu Y. Xie J. (2019a). Accurate and efficient P-value calculation via gaussian approximation: a novel monte-carlo method. *J. Am. Statist. Associat.* 114 384–392. 10.1080/01621459.2017.1407776 31130762 PMC6530914

[B49] Liu Y. Xie J. (2019b). Cauchy combination test: a powerful test with analytic p-value calculation under arbitrary dependency structures. *J. Am. Statist. Associat.* 115 1–29. 10.1080/01621459.2018.1554485 33012899 PMC7531765

[B50] Lopes-Ramos C. M. Chen C.-Y. Kuijjer M. L. Paulson J. N. Sonawane A. R. Fagny M. (2020). Sex differences in gene expression and regulatory networks across 29 human tissues. *Cell Rep.* 31:107795. 10.1016/j.celrep.2020.107795 PMC7898458 32579922

[B51] Lv W.-Q. Zhang X. Zhang Q. He J.-Y. Liu H.-M. Xia X. (2017). Novel common variants associated with body mass index and coronary artery disease detected using a pleiotropic cFDR method. *J. Mol. Cell. Cardiol.* 112 1–7. 10.1016/j.yjmcc.2017.08.011 28843344 PMC5812278

[B52] Manolio T. A. Collins F. S. Cox N. J. Goldstein D. B. Hindorff L. A. Hunter D. J. (2009). Finding the missing heritability of complex diseases. *Nature* 461 747–753. 19812666 10.1038/nature08494 PMC2831613

[B53] Manoussakis M. N. Georgopoulou C. Zintzaras E. Spyropoulou M. Stavropoulou A. Skopouli F. N. (2004). Sjögren’s syndrome associated with systemic lupus erythematosus: clinical and laboratory profiles and comparison with primary Sjögren’s syndrome. *Arthr. Rheumat.* 50 882–891. 15022331 10.1002/art.20093

[B54] Márquez A. Vidal-Bralo L. Rodríguez-Rodríguez L. González-Gay M. A. Balsa A. González-Álvaro I. (2017). A combined large-scale meta-analysis identifies COG6 as a novel shared risk locus for rheumatoid arthritis and systemic lupus erythematosus. *Ann. Rheum. Dis.* 76 286–294. 10.1136/annrheumdis-2016-209436 27193031

[B55] Meng Q. Qiu B. (2020). Exosomal MicroRNA-320a derived from mesenchymal stem cells regulates rheumatoid arthritis fibroblast-like synoviocyte activation by suppressing CXCL9 Expression. *Front. Physiol.* 11:441. 10.3389/fphys.2020.00441 32528301 PMC7264418

[B56] Michou L. Rat A.-C. Lasbleiz S. Bardin T. Cornélis F. (2008). Prevalence and distribution of autoimmune diseases in 368 rheumatoid arthritis families. *J. Rheumatol.* 35 790–796. 18381797

[B57] Nakatsu F. Messa M. Nández R. Czapla H. Zou Y. Strittmatter S. M. (2015). Sac2/INPP5F is an inositol 4-phosphatase that functions in the endocytic pathway. *J. Cell Biol.* 209 85–95. 10.1083/jcb.201409064 25869668 PMC4395491

[B58] Nicolae D. L. Gamazon E. Zhang W. Duan S. Dolan M. E. Cox N. J. (2010). Trait-associated SNPs are more likely to be eQTLs: annotation to enhance discovery from GWAS. *PLoS Genet.* 6:e1000888. 10.1371/journal.pgen.1000888 PMC2848547 20369019

[B59] Nicolaou S. A. Neumeier L. Takimoto K. Lee S. M. Duncan H. J. Kant S. K. (2010). Differential calcium signaling and Kv1.3 trafficking to the immunological synapse in systemic lupus erythematosus. *Cell Calc.* 47 19–28. 10.1016/j.ceca.2009.11.001 19959227 PMC2819652

[B60] Norén E. Almer S. Söderman J. (2017). Genetic variation and expression levels of tight junction genes identifies association between MAGI3 and inflammatory bowel disease. *BMC Gastroenterol.* 17:68. 10.1186/s12876-017-0620-y 28545409 PMC5445404

[B61] Okada Y. Kishikawa T. Sakaue S. Hirata J. (2017). Future directions of genomics research in rheumatic diseases. *Rheum. Dis. Clin.* 43 481–487. 10.1016/j.rdc.2017.04.009 28711147

[B62] Okada Y. Wu D. Trynka G. Raj T. Terao C. Ikari K. (2014). Genetics of rheumatoid arthritis contributes to biology and drug discovery. *Nature* 506 376–381. 10.1038/nature12873 24390342 PMC3944098

[B63] Oliva M. Muñoz-Aguirre M. Kim-Hellmuth S. Wucher V. Gewirtz A. D. H. Cotter D. J. (2020). The impact of sex on gene expression across human tissues. *Science* 369:eaba3066. 10.1126/science.aba3066 32913072 PMC8136152

[B64] Orozco G. Barrett J. C. Zeggini E. (2010). Synthetic associations in the context of genome-wide association scan signals. *Hum. Mol. Genet.* 19 R137–R144. 10.1093/hmg/ddq368 20805105 PMC2953742

[B65] Orozco G. Eyre S. Hinks A. Bowes J. Morgan A. W. Wilson A. G. (2011). Study of the common genetic background for rheumatoid arthritis and systemic lupus erythematosus. *Ann. Rheum. Dis.* 70 463–468. 21068098 10.1136/ard.2010.137174 PMC3033530

[B66] Orozco G. Sánchez E. González−Gay M. A. López−Nevot M. A. Torres B. Cáliz R. (2005). Association of a functional single−nucleotide polymorphism of PTPN22, encoding lymphoid protein phosphatase, with rheumatoid arthritis and systemic lupus erythematosus. *Arthr. Rheum. Off. J. Am. Coll. Rheumatol.* 52 219–224. 10.1002/art.20771 15641066

[B67] Pasaniuc B. Price A. L. (2016). Dissecting the genetics of complex traits using summary association statistics. *Nat. Rev. Genet.* 18 117–127. 10.1038/nrg.2016.142 27840428 PMC5449190

[B68] Peng C. Shen J. Lin X. Su K.-J. Greenbaum J. Zhu W. (2017). Genetic sharing with coronary artery disease identifies potential novel loci for bone mineral density. *Bone* 103 70–77. 10.1016/j.bone.2017.06.016 28651948 PMC5796548

[B69] Price A. L. Weale M. E. Patterson N. Myers S. R. Need A. C. Shianna K. V. (2008). Long-range LD can confound genome scans in admixed populations. *Am. J. Hum. Genet.* 83 132–135. 18606306 10.1016/j.ajhg.2008.06.005 PMC2443852

[B70] Price A. L. Zaitlen N. A. Reich D. Patterson N. (2010). New approaches to population stratification in genome-wide association studies. *Nat. Rev. Genet.* 11 459–463. 10.1038/nrg2813 20548291 PMC2975875

[B71] Ramos P. S. Criswell L. A. Moser K. L. Comeau M. E. Williams A. H. Pajewski N. M. (2011). A comprehensive analysis of shared loci between systemic lupus erythematosus (SLE) and sixteen autoimmune diseases reveals limited genetic overlap. *PLoS Genet.* 7:e1002406. 10.1371/journal.pgen.1002406 22174698 PMC3234215

[B72] Remmers E. F. Plenge R. M. Lee A. T. Graham R. R. Hom G. Behrens T. W. (2007). STAT4 and the risk of rheumatoid arthritis and systemic lupus erythematosus. *New Engl. J. Med.* 357 977–986. 17804842 10.1056/NEJMoa073003 PMC2630215

[B73] Rice K. (2010). A decision-theoretic formulation of fisher’s approach to testing. *Am. Statist.* 64 345–349. 10.1198/tast.2010.09060 12611515

[B74] Salaman M. R. (2003). A two-step hypothesis for the appearance of autoimmune disease. *Autoimmunity* 36 57–61. 10.1080/0891693031000068637 12820686

[B75] Schifano E. D. Epstein M. P. Bielak L. F. Jhun M. A. Kardia S. L. R. Peyser P. A. (2012). SNP set association analysis for familial data. *Genetic Epidemiol.* 36 797–810. 10.1002/gepi.21676 22968922 PMC3683469

[B76] Shimane K. Kochi Y. Horita T. Ikari K. Amano H. Hirakata M. (2010). The association of a nonsynonymous single−nucleotide polymorphism in TNFAIP3 with systemic lupus erythematosus and rheumatoid arthritis in the Japanese population. *Arthr. Rheum. Off. J. Am. Coll. Rheumatol.* 62 574–579. 10.1002/art.27190 20112363

[B77] Smeland O. B. Frei O. Shadrin A. O’Connell K. Fan C.-C. Bahrami S. (2020). Discovery of shared genomic loci using the conditional false discovery rate approach. *Hum. Genet.* 139 85–94. 31520123 10.1007/s00439-019-02060-2

[B78] Stastny P. (1978). Association of the B-cell alloantigen DRw4 with rheumatoid arthritis. *N. Engl. J. Med.* 298 869–871. 10.1056/nejm197804202981602 147420

[B79] Stojan G. Petri M. (2018). Epidemiology of systemic lupus erythematosus: an update. *Curr. Opin. Rheumatol.* 30 144–150. 10.1097/bor.0000000000000480 29251660 PMC6026543

[B80] Su Y. R. Di C. Bien S. Huang L. Dong X. Abecasis G. (2018). A mixed-effects model for powerful association tests in integrative functional genomics. *Am. J. Hum. Genet.* 102 904–919. 10.1016/j.ajhg.2018.03.019 29727690 PMC5986723

[B81] Sun R. Lin X. (2020). Genetic variant set-based tests using the generalized berk–jones statistic with application to a genome-wide association study of breast cancer. *J. Am. Statist. Associat.* 115 1079–1091. 10.1080/01621459.2019.1660170 33041403 PMC7539682

[B82] Suresh E. (2004). Diagnosis of early rheumatoid arthritis: what the non-specialist needs to know. *J. R. Soc. Med.* 97 421–424. 10.1258/jrsm.97.9.421 15340020 PMC1079582

[B83] Suryawanshi A. Tadagavadi R. K. Swafford D. Manicassamy S. (2016). Modulation of inflammatory responses by Wnt/β-catenin signaling in dendritic cells: a novel immunotherapy target for autoimmunity and cancer. *Front. Immunol.* 7:460. 10.3389/fimmu.2016.00460 27833613 PMC5081350

[B84] Suzuki K. Arumugam S. Yokoyama J. Kawauchi Y. Honda Y. Sato H. (2016). Pivotal role of carbohydrate sulfotransferase 15 in Fibrosis and mucosal healing in mouse colitis. *PLoS One* 11:e0158967. 10.1371/journal.pone.0158967 27410685 PMC4943596

[B85] Tam V. Patel N. Turcotte M. Bossé Y. Paré G. Meyre D. (2019). Benefits and limitations of genome-wide association studies. *Nat. Rev. Genet.* 20 467–484. 31068683 10.1038/s41576-019-0127-1

[B86] Tedeschi S. K. Bermas B. Costenbader K. H. (2013). Sexual disparities in the incidence and course of SLE and RA. *Clin. Immunol.* 149 211–218. 23578823 10.1016/j.clim.2013.03.003

[B87] Toro-Domínguez D. Carmona-Sáez P. Alarcón-Riquelme M. E. (2014). Shared signatures between rheumatoid arthritis, systemic lupus erythematosus and Sjögren’s syndrome uncovered through gene expression meta-analysis. *Arthr. Res. Ther.* 16:489. 10.1186/s13075-014-0489-x PMC4295333 25466291

[B88] van den Berg S. Vandenplas J. van Eeuwijk F. A. Lopes M. S. Veerkamp R. F. (2019). Significance testing and genomic inflation factor using high-density genotypes or whole-genome sequence data. *J. Anim. Breed Genet.* 136 418–429. 10.1111/jbg.12419 31215703 PMC6900143

[B89] Visscher P. M. Wray N. R. Zhang Q. Sklar P. McCarthy M. I. Brown M. A. (2017). 10 years of GWAS discovery: biology, function, and translation. *Am. J. Hum. Genet.* 101 5–22. 10.1016/j.ajhg.2017.06.005 28686856 PMC5501872

[B90] Wang W. Y. Barratt B. J. Clayton D. G. Todd J. A. (2005). Genome-wide association studies: theoretical and practical concerns. *Nat. Rev. Genet.* 6 109–118. 15716907 10.1038/nrg1522

[B91] Wang X. Lee S. Zhu X. Redline S. Lin X. (2013). GEE-based SNP Set association test for continuous and discrete traits in family-based association studies. *Genet. Epidemiol.* 37 778–786. 10.1002/gepi.21763 24166731 PMC4007511

[B92] Wilson D. (2019a). The harmonic mean p-value for combining dependent tests. *Proc. Natl. Acad. Sci. U.S.A.* 116 1195–1200. 10.1073/pnas.1814092116 30610179 PMC6347718

[B93] Wilson D. (2019b). *Harmonic Mean p-Values and Model Averaging by Mean Maximum Likelihood*. R package Version 3.0. Available online at: https://CRAN.R-project.org/package=harmonicmeanp

[B94] Wright H. L. Thomas H. B. Moots R. J. Edwards S. W. (2015). Interferon gene expression signature in rheumatoid arthritis neutrophils correlates with a good response to TNFi therapy. *Rheumatology (Oxford)* 54 188–193. 10.1093/rheumatology/keu299 25125592

[B95] Wu C. Pan W. (2018). Integrating eQTL data with GWAS summary statistics in pathway-based analysis with application to schizophrenia. *Genet. Epidemiol.* 42 303–316. 10.1002/gepi.22110 29411426 PMC5851843

[B96] Wu M. C. Kraft P. Epstein M. P. Taylor D. M. Chanock S. J. Hunter D. J. (2010). Powerful SNP-set analysis for case-control genome-wide association studies. *Am. J. Hum. Genet.* 86 929–942. 10.1016/j.ajhg.2010.05.002 20560208 PMC3032061

[B97] Wu M. C. Lee S. Cai T. Li Y. Boehnke M. Lin X. (2011). Rare-variant association testing for sequencing data with the sequence kernel association test. *Am. J. Hum. Genet.* 89 82–93. 21737059 10.1016/j.ajhg.2011.05.029 PMC3135811

[B98] Xiao L. Yuan Z. Jin S. Wang T. Huang S. Zeng P. (2020). Multiple-tissue integrative transcriptome-wide association studies discovered new genes associated with amyotrophic lateral sclerosis. *Front. Genet.* 11:587243. 10.3389/fgene.2020.587243 33329728 PMC7714931

[B99] Xu Z. Wu C. Wei P. Pan W. (2017). A powerful framework for integrating eQTL and GWAS summary data. *Genetics* 207 893–902. 10.1534/genetics.117.300270 28893853 PMC5676241

[B100] Xue H. Pan W. Alzheimer’s Disease Neuroimaging Initiative. (2020). Some statistical consideration in transcriptome-wide association studies. *Genet. Epidemiol.* 44 221–232. 10.1002/gepi.22274 31821608 PMC7064426

[B101] Zeng P. Zhao Y. Li H. Wang T. Chen F. (2015a). Permutation-based variance component test in generalized linear mixed model with application to multilocus genetic association study. *BMC Med. Res. Methodol.* 15:37. 10.1186/s12874-015-0030-1 PMC4410500 25897803

[B102] Zeng P. Zhao Y. Liu J. Liu L. Zhang L. Wang T. (2014a). Likelihood ratio tests in rare variant detection for continuous phenotypes. *Annal. Hum. Genet.* 78 320–332. 10.1111/ahg.12071 25117149

[B103] Zeng P. Zhao Y. Qian C. Zhang L. Zhang R. Gou J. (2015b). Statistical analysis for genome-wide association study. *J. Biomed. Res.* 29 285–297. 10.7555/jbr.29.20140007 26243515 PMC4547377

[B104] Zeng P. Zhao Y. Zhang L. Huang S. Chen F. (2014b). Rare variants detection with kernel machine learning based on likelihood ratio test. *PLoS One* 9:e93355. 10.1371/journal.pone.0093355 PMC3968153 24675868

[B105] Zhang J. Xie S. Gonzales S. Liu J. Wang X. (2020). A fast and powerful eQTL weighted method to detect genes associated with complex trait using GWAS summary data. *Genet. Epidemiol.* 44 550–563. 10.1002/gepi.22297 32350919

[B106] Zhang J. Zhao Z. Guo X. Guo B. Wu B. (2019). Powerful statistical method to detect disease-associated genes using publicly available genome-wide association studies summary data. *Genet. Epidemiol.* 43 941–951. 10.1002/gepi.22251 31392781

[B107] Zhernakova A. Van Diemen C. C. Wijmenga C. (2009). Detecting shared pathogenesis from the shared genetics of immune-related diseases. *Nat. Rev. Genet.* 10 43–55. 19092835 10.1038/nrg2489

[B108] Zhernakova A. Withoff S. Wijmenga C. (2013). Clinical implications of shared genetics and pathogenesis in autoimmune diseases. *Nat. Rev. Endocrinol.* 9 646–659. 10.1038/nrendo.2013.161 23959365

