# Supplementary material for: Detection of Genetic Overlap Between Rheumatoid Arthritis and Systemic Lupus Erythematosus Using GWAS Summary Statistics
Source: Front Genet. 2021 Mar 18;12:656545. doi: 10.3389/fgene.2021.656545 (PMC8012913; doi:10.3389/fgene.2021.656545)
Supplement: Supplementary file 1 [file Data_Sheet_1.docx]

Supplementary Material

# Supplementary Tables

Table S1. Common genetic loci that were associated with SLE and RA identified in previous genome-wide association studies

| gene | chr | position | |  | SNP | |
| --- | --- | --- | --- | --- | --- | --- |
|  |  | up | low |  | RA | SLE |
| *AP4B1* | 1 | 114,437,370 | 114,447,823 |  | rs1217393 | rs1217393 |
| *FCGR2A* | 1 | 161,475,220 | 161,493,803 |  | rs72717009, rs10494360 | rs1801274, rs6671847, rs12129787 |
| *FCGR2B* | 1 | 161,551,101 | 161,648,444 |  | rs75409195 | rs6697139, rs1801274 |
| *IL12RB2* | 1 | 67,773,047 | 67,862,583 |  | rs6659932 | rs1874791, rs6659932, rs10889681, rs3828069 |
| *IL6R* | 1 | 154,377,669 | 154,441,926 |  | rs2228145, rs8192284, rs2228145 | rs8192282 |
| *NCF2* | 1 | 183,524,698 | 183,560,011 |  | rs17849502 | rs17849501, rs13306575, rs10911363 |
| *PTPN22* | 1 | 114,356,433 | 114,414,381 |  | rs6679677, rs1217403, rs2476601 | rs6679677, rs2476601 |
| *PTPRC* | 1 | 198,607,801 | 198,726,545 |  | rs1932437, rs6696533 | rs34889541 |
| *TNFSF4* | 1 | 173,152,873 | 173,176,452 |  | rs61828284, rs2422345 | rs2205960, rs4916342, rs76413021, rs10753074, rs10912578, rs704840, rs2422345, rs6681482 |
| *CD28* | 2 | 204,571,198 | 204,602,557 |  | rs1980422 | rs4675354 |
| *LBH* | 2 | 30,454,397 | 30,546,596 |  | rs10175798, rs7579944 | rs7579944, rs17321999 |
| *LINC01185* | 2 | 60,825,132 | 60,881,314 |  | rs10203477 | rs1432296 |
| *NAB1* | 2 | 191,511,472 | 191,557,492 |  | rs744600, rs10931468 | rs744600 |
| *PLCL1* | 2 | 198,669,426 | 199,437,305 |  | rs6752643 | rs2196171, rs6738825 |
| *SPRED2* | 2 | 65,537,985 | 65,659,771 |  | rs1876518, rs934734, rs1858037, rs934734, rs11126034, rs11673987, rs6546146 | rs268134 |
| *STAT4* | 2 | 191,894,302 | 192,016,322 |  | rs7574865, rs13426947, rs6749371, rs11889341, rs13389408, rs10174238 | rs3821236, rs7601754, rs7574865, rs12612769, rs13389408, rs6736175, rs11889341, rs10174238, rs7582694, rs7568275 |
| *ABHD6* | 3 | 58,223,233 | 58,281,420 |  | rs73081554 | rs9311676 |
| *DNASE1L3* | 3 | 58,177,984 | 58,200,853 |  | rs13101828, rs73081554 | rs35677470 |
| *EOMES* | 3 | 27,757,440 | 27,764,206 |  | rs35677470, rs3806624, rs9880772 | rs427221 |
| *PXK* | 3 | 58,318,607 | 58,411,748 |  | rs13315591, rs73081554, rs35677470 | rs6445975, rs2176082, rs9311676, rs180977001, rs116165318, rs9852465 |
| *CLNK* | 4 | 10,488,019 | 10,686,489 |  | rs6032662 | rs4355385 |
| *DGKQ* | 4 | 952,675 | 980,683 |  | rs4938573 | rs13101828, rs3733345, rs4690229 |
| *IL2* | 4 | 123,372,625 | 123,377,880 |  | rs13119723, rs2104286, rs9826828, rs706778, rs45475795, rs62323881, rs10795791, rs3218251, rs3118470 | rs10795763, rs11724582, rs10905718, rs61839660 |
| *IL21* | 4 | 123,533,783 | 123,542,224 |  | rs13119723, rs45475795, rs62323881 | rs11724582 |
| *PTTG1* | 5 | 159,848,829 | 159,855,748 |  | rs4921283, rs2431098 | rs4921283, rs2431098 |
| *TNIP1* | 5 | 150,409,506 | 150,473,138 |  | rs6579837, rs4958880, rs1422673 | rs960709, rs6889239, rs10036748, rs7708392, rs4958880, rs10057690 |
| *ATG5* | 6 | 106,632,351 | 106,773,666 |  | rs9372120, rs6568431, rs9372120, rs802791 | rs548234, rs742108, rs3827644, rs6568431, rs802791, rs2299864, rs9373839 |
| *BACH2* | 6 | 90,636,248 | 91,006,627 |  | rs284515, rs284511, rs72928038 | rs12529935, rs597325 |
| *C6orf10* | 6 | 32,256,303 | 32,352,332 |  | rs9275406 | rs3130320, rs9267972 |
| *ETV7* | 6 | 36,322,419 | 36,356,164 |  | rs2234067 | rs916287 |
| *HLA-B* | 6 | 31,321,649 | 31,324,965 |  | rs2596565 |  |
| *HLA-DQA1* | 6 | 32,595,956 | 32,614,839 |  | rs6457617, rs9275406 | rs2647012, rs2187668 |
| *HLA-DQA2* | 6 | 32,709,119 | 32,714,992 |  | rs6457617, rs9275406, rs12525220 | rs2301271, rs2647012, rs2051549, rs9275572 |
| *HLA-DQB1* | 6 | 32,627,244 | 32,636,160 |  | rs9275406, rs12525220 | rs3129716, rs114092478 |
| *HLA-DRB1* | 6 | 32,546,546 | 32,557,625 |  | rs6457620, rs615672, rs7765379, rs660895, rs13192471, rs6910071, rs9268839, rs9271348, rs3104413, rs9269234, rs9268839, rs112112734 | rs9271100, rs9270984, rs3135394 |
| *OLIG3* | 6 | 137,813,336 | 137,815,531 |  | rs2230926, rs10499194, rs6920220, rs17264332 | rs2327832 |
| *TAGAP* | 6 | 159,455,500 | 159,466,184 |  | rs2451258, rs212389, rs212400, rs629326, rs2451258 | rs212407 |
| *TNFAIP3* | 6 | 138,188,351 | 138,204,449 |  | rs2230926, rs58721818, rs7752903, rs10499194, rs17264332, rs5029924, rs6920220 | rs5029939, rs2230926, rs10499197, rs6932056, rs5029937, rs6920220, rs5029924, rs58721818, rs77000060 |
| *IRF5* | 7 | 128,577,666 | 128,590,089 |  | rs10488631, rs4728142, rs3807306, rs12531711, rs3807306, rs3778753, rs10954214, rs4731532 | rs4728142, rs12537284, rs729302, rs13239597, rs113478424, rs3757387, rs10488631, rs2070197, rs12539741, rs10954214, rs35000415, rs12706861 |
| *JAZF1* | 7 | 27,870,192 | 28,220,362 |  | rs67250450 | rs1635852, rs12531540, rs849142, rs10254284, rs849142, rs10245867, rs702814 |
| *LIMK1* | 7 | 73,497,263 | 73,536,855 |  | rs193107685 | rs193107685 |
| *TNPO3* | 7 | 128,594,948 | 128,695,198 |  | rs12531711, rs13238352 | rs12537284, rs13239597, rs10488631, rs12539741, rs12531711, rs13238352, rs35000415, rs12706861, rs3757387 |
| *BLK* | 8 | 11,351,510 | 11,422,113 |  | rs1600249, rs2736340, rs922483, rs4840565, rs2736340, rs2736337 | rs2618476, rs2736340, rs7812879, rs13277113, rs2254546, rs2736340, rs1478897, rs2736332, rs2736345, rs2736337, rs2061831, rs2736336, rs2618444, rs12548184 |
| *MIR1208* | 8 | 129,162,362 | 129,162,434 |  | rs6651252 | rs13280095 |
| *PVT1* | 8 | 128,806,779 | 129,113,499 |  | rs1516971, rs6651252 | rs12156002 |
| *ARID5B* | 10 | 63,661,059 | 63,856,703 |  | rs10821944, rs71508903, rs12764378 | rs4948496, rs10995092 |
| *GATA3* | 10 | 8,095,567 | 8,117,161 |  | rs3824660, rs3781094, rs2275806, rs3802604 | rs7090925 |
| *IL2RA* | 10 | 6,052,652 | 6,104,333 |  | rs2104286, rs706778, rs10795791 | rs10795763, rs10905718, rs61839660 |
| *RTKN2* | 10 | 63,942,794 | 64,028,466 |  | rs3125734, rs6479800 | rs4948496 |
| *WDFY4* | 10 | 49,892,921 | 50,191,001 |  | rs2671692 | rs1913517, rs7097397, rs877819, rs2663052, rs2928402, rs10857635, rs2663054 |
| *DDX6* | 11 | 118,620,034 | 118,661,858 |  | rs7993214, rs10892279, rs11217037 | rs503425, rs10892286 |
| *ETS1* | 11 | 128,328,656 | 128,457,437 |  | rs3806624, rs4937362, rs73013527, rs4936059, rs73013527 | rs6590330, rs1128334, rs6590330, rs7941765, rs1128334, rs12574073, rs1946007 |
| *FLI1* | 11 | 128,555,046 | 128,683,161 |  | rs4937362 | rs7941765 |
| *PTPN11* | 12 | 112,856,155 | 112,947,717 |  | rs10774624, rs11066301 | rs11066301 |
| *SH2B3* | 12 | 111,843,752 | 111,889,427 |  | rs653178, rs3184504, rs10774624, rs11066188 | rs597808, rs10774625 |
| *COG6* | 13 | 40,229,764 | 40,365,802 |  | rs13142500, rs9603616, rs9603616 | rs9603612 |
| *AHNAK2* | 14 | 105,403,581 | 105,444,694 |  | rs2582532 | rs1048257 |
| *RAD51B* | 14 | 68,286,496 | 69,196,935 |  | rs1950897, rs911263, rs140825531 | rs4902562, rs911263 |
| *RASGRP1* | 15 | 38,780,304 | 38,857,776 |  | rs8032939 | rs1874252 |
| *IRF8* | 16 | 85,932,409 | 85,956,197 |  | rs2280381, rs13330176, rs35929052 | rs12711490, rs10521318, rs13332649, rs11644034, rs12444486, rs35929052, rs11117433 |
| *GSDMB* | 17 | 38,060,848 | 38,077,313 |  | rs12936409, rs2872507 | rs2941509 |
| *IKZF3* | 17 | 37,913,968 | 38,020,441 |  | rs12936409, rs2305480, rs2872507, rs59716545 | rs9303277, rs2941509, rs143123127, rs2941509 |
| *MED1* | 17 | 37,560,538 | 37,607,521 |  | rs1877030 | rs11655550 |
| *ICAM3* | 19 | 10,444,454 | 10,450,345 |  | rs7258015, rs34536443 | rs7258015 |
| *ILF3* | 19 | 10,764,937 | 10,803,093 |  | rs147622113 | rs147622113 |
| *PRR12* | 19 | 50,094,912 | 50,129,695 |  | rs76246107 | rs76246107 |
| *TYK2* | 19 | 10,461,209 | 10,491,352 |  | rs34536443, rs7258015, rs11085725 | rs11085727, rs2304256, rs7258015, rs11085725, rs34536443, rs34725611 |
| *CD40* | 20 | 44,746,911 | 44,758,502 |  | rs3087243, rs4810485, rs4239702, rs1883832, rs4810485 | rs6131014, rs4810485 |
| *UBE2L3* | 22 | 21,903,736 | 21,978,323 |  | rs2298428, rs11089637, rs4821124, rs2283790 | rs5754217, rs131654, rs3747093, rs7444, rs2283790, rs131658 |
| *YDJC* | 22 | 21,982,378 | 21,984,353 |  | rs11089637, rs5754467 | rs7444, rs5754467 |
| *HCFC1* | X | 153,947,553 | 153,971,807 |  | rs13397 | rs1734787 |
| *IRAK1* | X | 154,010,500 | 154,019,980 |  | rs5987194, rs13397 | rs5986948, rs1734787, rs1059702, rs2269368 |
| *TMEM187* | X | 153,972,327 | 153,983,195 |  | rs13397 | rs5986948, rs1734787 |

Table S2. Summary information of tissues in GTEx

| tissues | *N* | Number of gene | |
| --- | --- | --- | --- |
|  |  | SLE | RA |
| adipose subcutaneous | 385 | 8041 | 8058 |
| adipose visceral omentum | 313 | 6393 | 6413 |
| adrenal gland | 175 | 4477 | 4484 |
| artery aorta | 267 | 6468 | 6479 |
| artery coronary | 152 | 3378 | 3387 |
| artery tibial | 388 | 8022 | 8042 |
| brain amygdala | 88 | 2289 | 2295 |
| brain anterior cingulate cortex ba24 | 109 | 3205 | 3213 |
| brain caudate basal ganglia | 144 | 4054 | 4063 |
| brain cerebellar hemisphere | 125 | 4628 | 4632 |
| brain cerebellum | 154 | 5934 | 5943 |
| brain cortex | 136 | 4203 | 4214 |
| brain frontal cortex ba9 | 118 | 3481 | 3488 |
| brain hippocampus | 111 | 2722 | 2728 |
| brain hypothalamus | 108 | 2734 | 2742 |
| brain nucleus accumbens basa ganglia | 130 | 3517 | 3529 |
| brain putamen basal ganglia | 111 | 3078 | 3088 |
| brain spinal cord cervical c-1 | 83 | 2435 | 2441 |
| brain substantia nigra | 80 | 1975 | 1979 |
| cells EBV-transformed lymphocytes | 117 | 2938 | 2946 |
| cells transformed fibroblasts | 300 | 7180 | 7195 |
| colon sigmoid | 203 | 5065 | 5082 |
| colon transverse | 246 | 5437 | 5449 |
| esophagus gastroesophageal junction | 213 | 5092 | 5109 |
| esophagus mucosa | 358 | 7965 | 7984 |
| esophagus muscularis | 335 | 7535 | 7559 |
| heart atrial appendage | 264 | 5808 | 5823 |
| heart left ventricle | 272 | 5207 | 5225 |
| liver | 153 | 3235 | 3248 |
| lung | 383 | 7722 | 7751 |
| minor salivary gland | 85 | 2169 | 2176 |
| muscle skeletal | 491 | 7326 | 7342 |
| nerve tibial | 361 | 9162 | 9191 |
| pancreas | 220 | 5192 | 5199 |
| pituitary | 157 | 4629 | 4632 |
| skin not sun exposed suprapubic | 335 | 7445 | 7459 |
| skin sun exposed lower leg | 414 | 8876 | 8902 |
| small intestine terminal ileum | 122 | 2992 | 3001 |
| spleen | 146 | 4466 | 4475 |
| stomach | 237 | 4644 | 4657 |
| thyroid | 399 | 9392 | 9412 |
| whole blood | 369 | 6103 | 6118 |

Note: *N*: the total number of sample size for each GTEx tissue; gene numbers: the number of converged cis-heritable genes when estimating the heritability with cis-SNPs. SLE: systemic lupus erythematosus; RA: rheumatoid arthritis.

Table S3. Genomic inflation factor of various tissues for RA and SLE

| tissue | RA | SLE |
| --- | --- | --- |
| Adipose Subcutaneous | 1.113 | 1.466 |
| Adipose Visceral Omentum | 1.114 | 1.474 |
| Adrenal Gland | 1.077 | 1.446 |
| Artery Aorta | 1.104 | 1.548 |
| Artery Coronary | 1.051 | 1.382 |
| Artery Tibial | 1.142 | 1.498 |
| Brain Amygdala | 0.958 | 1.405 |
| Brain Anterior cingulate cortex BA24 | 0.986 | 1.459 |
| Brain Caudate basal ganglia | 1.041 | 1.384 |
| Brain Cerebellar Hemisphere | 1.056 | 1.482 |
| Brain Cerebellum | 1.069 | 1.412 |
| Brain Cortex | 1.022 | 1.443 |
| Brain Frontal Cortex BA9 | 1.027 | 1.276 |
| Brain Hippocampus | 0.938 | 1.384 |
| Brain Hypothalamus | 0.964 | 1.348 |
| Brain Nucleus accumbens basal ganglia | 1.091 | 1.487 |
| Brain Putamen basal ganglia | 1.000 | 1.340 |
| Brain Spinal cord cervical c-1 | 0.861 | 1.279 |
| Brain Substantia nigra | 0.876 | 1.358 |
| Cells EBV-transformed lymphocytes | 0.992 | 1.365 |
| Cells Transformed fibroblasts | 1.086 | 1.478 |
| Colon Sigmoid | 1.111 | 1.502 |
| Colon Transverse | 1.073 | 1.467 |
| Esophagus Gastroesophageal Junction | 1.138 | 1.461 |
| Esophagus Mucosa | 1.137 | 1.515 |
| Esophagus Muscularis | 1.125 | 1.464 |
| Heart Atrial Appendage | 1.053 | 1.446 |
| Heart Left Ventricle | 1.066 | 1.475 |
| Liver | 0.986 | 1.379 |
| Lung | 1.098 | 1.509 |
| Minor Salivary Gland | 0.993 | 1.470 |
| Muscle Skeletal | 1.101 | 1.521 |
| Nerve Tibial | 1.092 | 1.420 |
| Pancreas | 1.162 | 1.473 |
| Pituitary | 1.034 | 1.454 |
| Skin Not Sun Exposed Suprapubic | 1.134 | 1.463 |
| Skin Sun Exposed Lower leg | 1.169 | 1.479 |
| Small Intestine Terminal Ileum | 1.010 | 1.311 |
| Spleen | 1.056 | 1.410 |
| Stomach | 1.124 | 1.484 |
| Thyroid | 1.124 | 1.531 |
| Whole Blood | 1.106 | 1.502 |

Note: SLE: systemic lupus erythematosus; RA: rheumatoid arthritis.

Table S4. Genes associated with RA detected by cFDR

| Gene | chr | position | HMP | |  | cFDR | | ccFDR |
| --- | --- | --- | --- | --- | --- | --- | --- | --- |
|  |  |  | RA | SLE |  | RA | SLE |  |
| *CCBL2* | 1 | 89,401,456-89,458,636 | 6.47E-04 | 4.11E-01 |  | 4.83E-02 | 1.08E+00 | 1.08E+00 |
| *CCDC18* | 1 | 93,645,476-93,744,287 | 1.74E-03 | 1.47E-02 |  | 3.38E-02 | 1.94E-01 | 1.94E-01 |
| *INPP5B* | 1 | 38,326,369-38,412,729 | 3.91E-16 | 5.64E-03 |  | 1.58E-14 | 2.54E-02 | 2.54E-02 |
| *RSBN1* | 1 | 114,304,454-114,355,098 | 4.73E-24 | 2.75E-01 |  | 1.06E-21 | 3.14E-01 | 3.14E-01 |
| *SNRPE* | 1 | 203,830,731-203,839,678 | 6.39E-05 | 9.41E-01 |  | 2.80E-02 | 1.03E+00 | 1.03E+00 |
| *TMED5* | 1 | 93,615,299-93,646,285 | 3.66E-03 | 5.45E-03 |  | 3.66E-02 | 9.54E-02 | 9.54E-02 |
| *AC097523.2* | 2 | 139,045,599-139,046,363 | 3.68E-05 | 6.19E-01 |  | 4.87E-03 | 9.90E-01 | 9.90E-01 |
| *PSD4* | 2 | 113,914,902-113,960,814 | 1.07E-04 | 1.09E-01 |  | 4.66E-03 | 3.10E-01 | 3.10E-01 |
| *RP11-181E10.3* | 2 | 111,968,572-111,970,053 | 3.32E-05 | 8.27E-01 |  | 1.15E-02 | 1.01E+00 | 1.01E+00 |
| *OR5K2* | 3 | 98,216,448-98,217,496 | 1.42E-02 | 9.12E-04 |  | 4.63E-02 | 2.37E-02 | 4.63E-02 |
| *PROS1* | 3 | 93,591,881-93,692,910 | 1.07E-03 | 1.61E-01 |  | 3.98E-02 | 5.25E-01 | 5.25E-01 |
| *SLC10A4* | 4 | 48,485,360-48,491,213 | 1.52E-04 | 5.49E-01 |  | 1.57E-02 | 1.06E+00 | 1.06E+00 |
| *DAP* | 5 | 10,679,342-10,761,384 | 2.88E-06 | 5.53E-01 |  | 4.91E-04 | 8.13E-01 | 8.13E-01 |
| *EIF3KP1* | 5 | 103,032,376-103,033,031 | 1.94E-05 | 6.55E-02 |  | 1.01E-03 | 2.16E-01 | 2.16E-01 |
| *PPIP5K2* | 5 | 102,455,853-102,538,937 | 4.93E-04 | 5.93E-02 |  | 1.68E-02 | 3.05E-01 | 3.05E-01 |
| *ABT1* | 6 | 26,597,180-26,600,278 | 6.58E-05 | 5.21E-01 |  | 7.17E-03 | 9.59E-01 | 9.59E-01 |
| *BAK1* | 6 | 33,540,324-33,548,070 | 3.50E-29 | 3.42E-01 |  | 1.28E-26 | 3.42E-01 | 3.42E-01 |
| *BTN2A3P* | 6 | 26,421,619-26,431,928 | 2.12E-04 | 4.98E-01 |  | 2.03E-02 | 1.07E+00 | 1.07E+00 |
| *C6orf106* | 6 | 34,555,065-34,664,636 | 8.74E-04 | 5.65E-02 |  | 2.50E-02 | 3.07E-01 | 3.07E-01 |
| *FGFR1OP* | 6 | 167,412,670-167,455,906 | 1.10E-06 | 1.03E-01 |  | 1.03E-04 | 2.95E-01 | 2.95E-01 |
| *ITPR3* | 6 | 33,588,522-33,664,351 | 9.83E-27 | 4.60E-04 |  | 2.85E-25 | 2.76E-03 | 2.76E-03 |
| *LEMD2* | 6 | 33,738,979-33,756,913 | 1.16E-13 | 7.17E-01 |  | 5.98E-11 | 7.89E-01 | 7.89E-01 |
| *RPS10* | 6 | 34,385,231-34,393,902 | 1.70E-29 | 1.42E-01 |  | 8.15E-27 | 1.42E-01 | 1.42E-01 |
| *SCGN* | 6 | 25,652,464-25,702,011 | 3.17E-04 | 5.25E-01 |  | 2.90E-02 | 1.10E+00 | 1.10E+00 |
| *HMGB1P41* | 8 | 81,724,377-81,724,816 | 2.12E-04 | 3.64E-01 |  | 1.65E-02 | 8.59E-01 | 8.59E-01 |
| *FAM205A* | 9 | 34,723,052-34,729,464 | 4.37E-07 | 4.53E-01 |  | 7.87E-05 | 6.62E-01 | 6.62E-01 |
| *LCN2* | 9 | 130,911,350-130,915,734 | 3.48E-04 | 6.77E-01 |  | 3.72E-02 | 1.26E+00 | 1.26E+00 |
| *PLEKHA1* | 10 | 124,134,173-124,191,867 | 5.35E-05 | 9.84E-01 |  | 2.73E-02 | 1.01E+00 | 1.01E+00 |
| *DCPS* | 11 | 126,173,647-126,215,644 | 8.29E-04 | 4.03E-02 |  | 2.24E-02 | 2.60E-01 | 2.60E-01 |
| *FADS2* | 11 | 61,560,452-61,634,826 | 7.20E-06 | 3.95E-01 |  | 8.85E-04 | 6.73E-01 | 6.73E-01 |
| *PPP1R14B* | 11 | 64,011,956-64,014,413 | 2.96E-05 | 3.48E-02 |  | 1.14E-03 | 1.56E-01 | 1.56E-01 |
| *GATC* | 12 | 120,884,241-120,899,389 | 8.46E-05 | 9.81E-01 |  | 3.99E-02 | 1.05E+00 | 1.05E+00 |
| *IKZF4* | 12 | 56,401,443-56,432,219 | 8.86E-08 | 1.06E-01 |  | 9.72E-06 | 2.58E-01 | 2.58E-01 |
| *METTL21B* | 12 | 58,165,275-58,176,324 | 1.03E-06 | 8.20E-01 |  | 5.97E-04 | 1.00E+00 | 1.00E+00 |
| *RPS26* | 12 | 56,435,637-56,438,116 | 2.58E-07 | 1.08E-01 |  | 2.52E-05 | 2.44E-01 | 2.44E-01 |
| *SUOX* | 12 | 56,390,964-56,400,425 | 6.24E-12 | 5.51E-01 |  | 1.81E-09 | 7.17E-01 | 7.17E-01 |
| *TSFM* | 12 | 58,176,372-58,201,854 | 4.80E-07 | 8.47E-01 |  | 3.41E-04 | 9.96E-01 | 9.96E-01 |
| *PSTPIP1* | 15 | 77,285,700-77,329,673 | 7.75E-04 | 3.81E-02 |  | 2.16E-02 | 2.60E-01 | 2.60E-01 |
| *BOLA2* | 16 | 29,464,914-29,466,285 | 7.99E-05 | 9.10E-01 |  | 3.09E-02 | 1.07E+00 | 1.07E+00 |
| *CDC37P1* | 16 | 28,711,615-28,712,861 | 1.43E-04 | 1.81E-01 |  | 7.38E-03 | 4.36E-01 | 4.36E-01 |
| *CORO1A* | 16 | 30,194,148-30,200,397 | 3.19E-05 | 7.14E-01 |  | 5.81E-03 | 9.78E-01 | 9.78E-01 |
| *LPCAT2* | 16 | 55,542,910-55,620,582 | 9.19E-05 | 7.67E-01 |  | 2.07E-02 | 1.13E+00 | 1.13E+00 |
| *RNF40* | 16 | 30,773,066-30,787,628 | 2.77E-08 | 5.33E-02 |  | 2.42E-06 | 1.60E-01 | 1.60E-01 |
| *RP11-1348G14.6* | 16 | 28,761,280-28,761,916 | 4.15E-05 | 1.70E-01 |  | 2.25E-03 | 3.57E-01 | 3.57E-01 |
| *RP11-231C14.4* | 16 | 29,495,010-29,517,320 | 1.64E-05 | 9.93E-01 |  | 1.22E-02 | 9.93E-01 | 9.93E-01 |
| *RP11-2C24.5* | 16 | 30,832,389-30,833,431 | 1.86E-02 | 2.34E-16 |  | 3.72E-02 | 8.03E-14 | 3.72E-02 |
| *SH2B1* | 16 | 28,857,921-28,885,526 | 5.03E-05 | 8.23E-01 |  | 1.56E-02 | 1.04E+00 | 1.04E+00 |
| *TUFM* | 16 | 28,853,732-28,857,729 | 3.85E-04 | 6.94E-01 |  | 4.23E-02 | 1.26E+00 | 1.26E+00 |
| *KCNH4* | 17 | 40,308,910-40,333,296 | 1.44E-03 | 8.62E-02 |  | 4.40E-02 | 4.19E-01 | 4.19E-01 |
| *KRT223P* | 17 | 38,873,487-38,878,184 | 2.08E-04 | 7.11E-01 |  | 3.01E-02 | 1.21E+00 | 1.21E+00 |
| *RAB5C* | 17 | 40,276,994-40,307,035 | 7.38E-04 | 5.95E-02 |  | 2.21E-02 | 3.01E-01 | 3.01E-01 |
| *SHPK* | 17 | 3,511,556-3,539,616 | 1.11E-05 | 9.35E-01 |  | 6.95E-03 | 1.00E+00 | 1.00E+00 |
| *RP11-973H7.1* | 18 | 12,774,650-12,775,922 | 8.91E-13 | 9.83E-01 |  | 1.62E-09 | 9.83E-01 | 9.83E-01 |
| *CILP2* | 19 | 19,649,074-19,657,468 | 1.25E-03 | 9.37E-02 |  | 3.92E-02 | 4.09E-01 | 4.09E-01 |
| *CTD-3105H18.4* | 19 | 12,490,560-12,494,501 | 1.37E-02 | 2.79E-04 |  | 4.45E-02 | 9.82E-03 | 4.45E-02 |
| *TM6SF2* | 19 | 19,375,174-19,384,074 | 6.59E-05 | 8.45E-01 |  | 2.12E-02 | 1.07E+00 | 1.07E+00 |
| *TOMM40* | 19 | 45,394,477-45,406,935 | 3.46E-04 | 5.47E-01 |  | 3.20E-02 | 1.16E+00 | 1.16E+00 |
| *C1QTNF6* | 22 | 37,576,207-37,595,425 | 3.51E-04 | 5.91E-01 |  | 3.13E-02 | 1.15E+00 | 1.15E+00 |
| *XPNPEP3* | 22 | 41,253,081-41,368,585 | 3.40E-05 | 9.34E-01 |  | 1.69E-02 | 1.04E+00 | 1.04E+00 |
| *MAGI3* | 1 | 113,933,371-114,228,545 | 1.81E-30 | 1.60E-02 |  | 3.02E-28 | 1.60E-02 | 1.60E-02 |
| *PADI4* | 1 | 17,634,690-17,690,499 | 4.77E-08 | 9.02E-01 |  | 5.21E-05 | 1.03E+00 | 1.03E+00 |
| *HSPA7* | 1 | 161,576,081-161,578,007 | 1.72E-06 | 6.96E-01 |  | 4.26E-04 | 9.82E-01 | 9.82E-01 |
| *FCRL3* | 1 | 157,646,271-157,670,775 | 1.82E-05 | 7.13E-01 |  | 4.06E-03 | 1.04E+00 | 1.04E+00 |
| *PUS10* | 2 | 61,169,104-61,245,389 | 2.50E-09 | 7.23E-01 |  | 1.20E-06 | 8.44E-01 | 8.44E-01 |
| *INPP1* | 2 | 191,208,196-191,236,391 | 7.07E-03 | 5.79E-05 |  | 4.24E-02 | 3.86E-03 | 4.24E-02 |
| *RFTN2* | 2 | 198,435,524-198,540,769 | 2.15E-05 | 3.21E-02 |  | 1.05E-03 | 1.82E-01 | 1.82E-01 |
| *PDHB* | 3 | 58,413,357-58,419,584 | 2.51E-05 | 5.66E-04 |  | 2.60E-04 | 6.60E-03 | 6.60E-03 |
| *CLIP2* | 7 | 73,703,803-73,820,273 | 4.88E-03 | 7.32E-05 |  | 3.09E-02 | 4.03E-03 | 3.09E-02 |
| *IRF5* | 7 | 128,577,666-128,590,089 | 5.45E-04 | 4.27E-11 |  | 2.72E-03 | 3.12E-09 | 2.72E-03 |
| *COG6* | 13 | 40,229,764-40,365,802 | 5.04E-06 | 8.89E-01 |  | 2.93E-03 | 9.95E-01 | 9.95E-01 |
| *ORMDL3* | 17 | 38,077,294-38,083,854 | 3.26E-06 | 5.56E-02 |  | 2.10E-04 | 2.07E-01 | 2.07E-01 |
| *RP11-387H17.4* | 17 | 38,083,995-38,095,854 | 4.14E-05 | 1.06E-04 |  | 4.14E-04 | 2.17E-03 | 2.17E-03 |
| *ICAM5* | 19 | 10,400,655-10,407,453 | 1.90E-03 | 1.57E-04 |  | 1.09E-02 | 4.33E-03 | 1.09E-02 |
| *TYK2* | 19 | 10,461,209-10,491,352 | 1.01E-06 | 2.57E-05 |  | 1.61E-05 | 5.39E-04 | 5.39E-04 |
| *CD40* | 20 | 44,746,911-44,758,502 | 2.60E-29 | 1.50E-01 |  | 6.47E-27 | 1.50E-01 | 1.50E-01 |
| *CCDC116* | 22 | 21,987,007-21,991,616 | 1.06E-02 | 3.38E-16 |  | 3.18E-02 | 8.52E-14 | 3.18E-02 |

Note: Top 59 lines may be newly identified potential pleiotropy genes with RA. The statistics under HMP and cFDR is the p value after HMP and cFDR, respectively. RA: rheumatoid arthritis, SLE: systemic lupus erythematosus.

Table S5. Genes associated with SLE detected by cFDR

| Gene | chr | position | HMP  p_SLE | |  | cFDR | | ccFDR |
| --- | --- | --- | --- | --- | --- | --- | --- | --- |
|  |  |  | RA | SLE |  | RA | SLE |  |
| *INPP5B* | 1 | 38,326,369-38,412,729 | 3.91E-16 | 5.64E-03 |  | 1.58E-14 | 2.54E-02 | 2.54E-02 |
| *OR5K2* | 3 | 98,216,448-98,217,496 | 1.42E-02 | 9.12E-04 |  | 4.63E-02 | 2.37E-02 | 4.63E-02 |
| *RP13-685P2.7* | 3 | 129,064,254-129,065,007 | 9.60E-01 | 2.41E-14 |  | 9.60E-01 | 1.15E-10 | 9.60E-01 |
| *SKP1* | 5 | 133,492,082-133,561,762 | 5.24E-02 | 1.15E-03 |  | 1.26E-01 | 4.40E-02 | 1.26E-01 |
| *BTN2A2* | 6 | 26,383,324-26,395,102 | 9.57E-01 | 3.48E-07 |  | 1.05E+00 | 6.55E-04 | 1.05E+00 |
| *BTN3A3* | 6 | 26,440,700-26,453,643 | 1.26E-01 | 4.89E-08 |  | 2.27E-01 | 1.11E-05 | 2.27E-01 |
| *ITPR3* | 6 | 33,588,522-33,664,351 | 9.83E-27 | 4.60E-04 |  | 2.85E-25 | 2.76E-03 | 2.76E-03 |
| *SNRPC* | 6 | 34,725,183-34,741,571 | 2.38E-02 | 1.68E-04 |  | 6.35E-02 | 7.58E-03 | 6.35E-02 |
| *TAF11* | 6 | 34,845,555-34,855,866 | 4.38E-01 | 1.81E-04 |  | 6.44E-01 | 2.74E-02 | 6.44E-01 |
| *TCP11* | 6 | 35,085,848-35,116,387 | 9.31E-01 | 2.47E-05 |  | 1.07E+00 | 3.23E-02 | 1.07E+00 |
| *UHRF1BP1* | 6 | 34,759,857-34,850,915 | 8.30E-02 | 1.12E-04 |  | 1.58E-01 | 8.78E-03 | 1.58E-01 |
| *CD44* | 11 | 35,160,417-35,253,949 | 8.04E-01 | 3.25E-08 |  | 9.19E-01 | 4.60E-05 | 9.19E-01 |
| *HRAS* | 11 | 532,242-537,287 | 4.78E-01 | 1.74E-06 |  | 6.21E-01 | 4.82E-04 | 6.21E-01 |
| *TMEM80* | 11 | 695,616-705,028 | 9.15E-01 | 2.09E-06 |  | 1.07E+00 | 2.78E-03 | 1.07E+00 |
| *FUS* | 16 | 31,191,431-31,203,127 | 9.15E-02 | 1.14E-04 |  | 1.68E-01 | 8.65E-03 | 1.68E-01 |
| *RP11-2C24.5* | 16 | 30,832,389-30,833,431 | 1.86E-02 | 2.34E-16 |  | 3.72E-02 | 8.03E-14 | 3.72E-02 |
| *ZNF843* | 16 | 31,445,569-31,454,346 | 3.32E-01 | 5.42E-17 |  | 3.32E-01 | 1.14E-13 | 3.32E-01 |
| *CTD-3105H18.4* | 19 | 12,490,560-12,494,501 | 1.37E-02 | 2.79E-04 |  | 4.45E-02 | 9.82E-03 | 4.45E-02 |
| *PLEKHJ1* | 19 | 2,233,155-2,236,319 | 3.34E-01 | 4.15E-04 |  | 5.20E-01 | 4.89E-02 | 5.20E-01 |
| *MAGI3* | 1 | 113,933,371-114,228,545 | 1.81E-30 | 1.60E-02 |  | 3.02E-28 | 1.60E-02 | 1.60E-02 |
| *INPP1* | 2 | 191,208,196-191,236,391 | 7.07E-03 | 5.79E-05 |  | 4.24E-02 | 3.86E-03 | 4.24E-02 |
| *NAB1* | 2 | 191,511,472-191,557,492 | 3.77E-02 | 1.21E-08 |  | 6.60E-02 | 1.59E-06 | 6.60E-02 |
| *PDHB* | 3 | 58,413,357-58,419,584 | 2.51E-05 | 5.66E-04 |  | 2.60E-04 | 6.60E-03 | 6.60E-03 |
| *PXK* | 3 | 58,318,607-58,411,748 | 7.83E-02 | 2.76E-07 |  | 1.57E-01 | 4.63E-05 | 1.57E-01 |
| *CLIP2* | 7 | 73,703,803-73,820,273 | 4.88E-03 | 7.32E-05 |  | 3.09E-02 | 4.03E-03 | 3.09E-02 |
| *IRF5* | 7 | 128,577,666-128,590,089 | 5.45E-04 | 4.27E-11 |  | 2.72E-03 | 3.12E-09 | 2.72E-03 |
| *RP11-110I1.14* | 11 | 118,935,974-118,936,388 | 2.30E-01 | 4.98E-04 |  | 3.83E-01 | 4.55E-02 | 3.83E-01 |
| *RP11-387H17.4* | 17 | 38,083,995-38,095,854 | 4.14E-05 | 1.06E-04 |  | 4.14E-04 | 2.17E-03 | 2.17E-03 |
| *ICAM5* | 19 | 10,400,655-10,407,453 | 1.90E-03 | 1.57E-04 |  | 1.09E-02 | 4.33E-03 | 1.09E-02 |
| *TYK2* | 19 | 10,461,209-10,491,352 | 1.01E-06 | 2.57E-05 |  | 1.61E-05 | 5.39E-04 | 5.39E-04 |
| *CCDC116* | 22 | 21,987,007-21,991,616 | 1.06E-02 | 3.38E-16 |  | 3.18E-02 | 8.52E-14 | 3.18E-02 |
| *UBE2L3* | 22 | 21,903,736-21,978,323 | 5.08E-02 | 1.18E-06 |  | 1.22E-01 | 1.49E-04 | 1.22E-01 |
| *YDJC* | 22 | 21,982,378-21,984,353 | 4.50E-01 | 4.26E-09 |  | 5.40E-01 | 2.25E-06 | 5.40E-01 |

Note: Top 19 lines may be newly identified potential pleiotropy genes with SLE. The statistics under HMP and cFDR is the p value after HMP and cFDR, respectively. RA: rheumatoid arthritis, SLE: systemic lupus erythematosus.

# Supplementary Figures


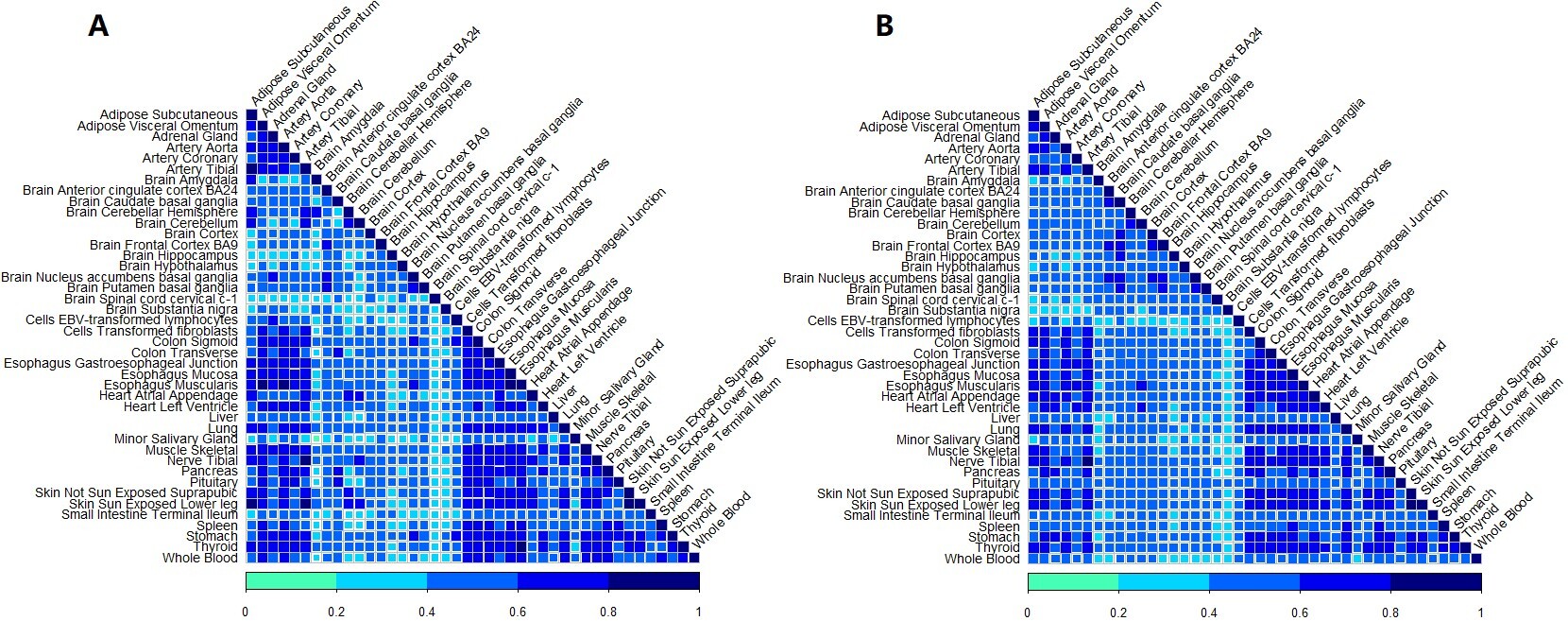


**Supplementary Figure 1.** Correlation among -log10 P values from different tissues in the eQTL-weighted association analysis for RA (A) and SLE (B). The average across-tissue correlations are 0.523 and 0.522 for RA and SLE, respectively.

**
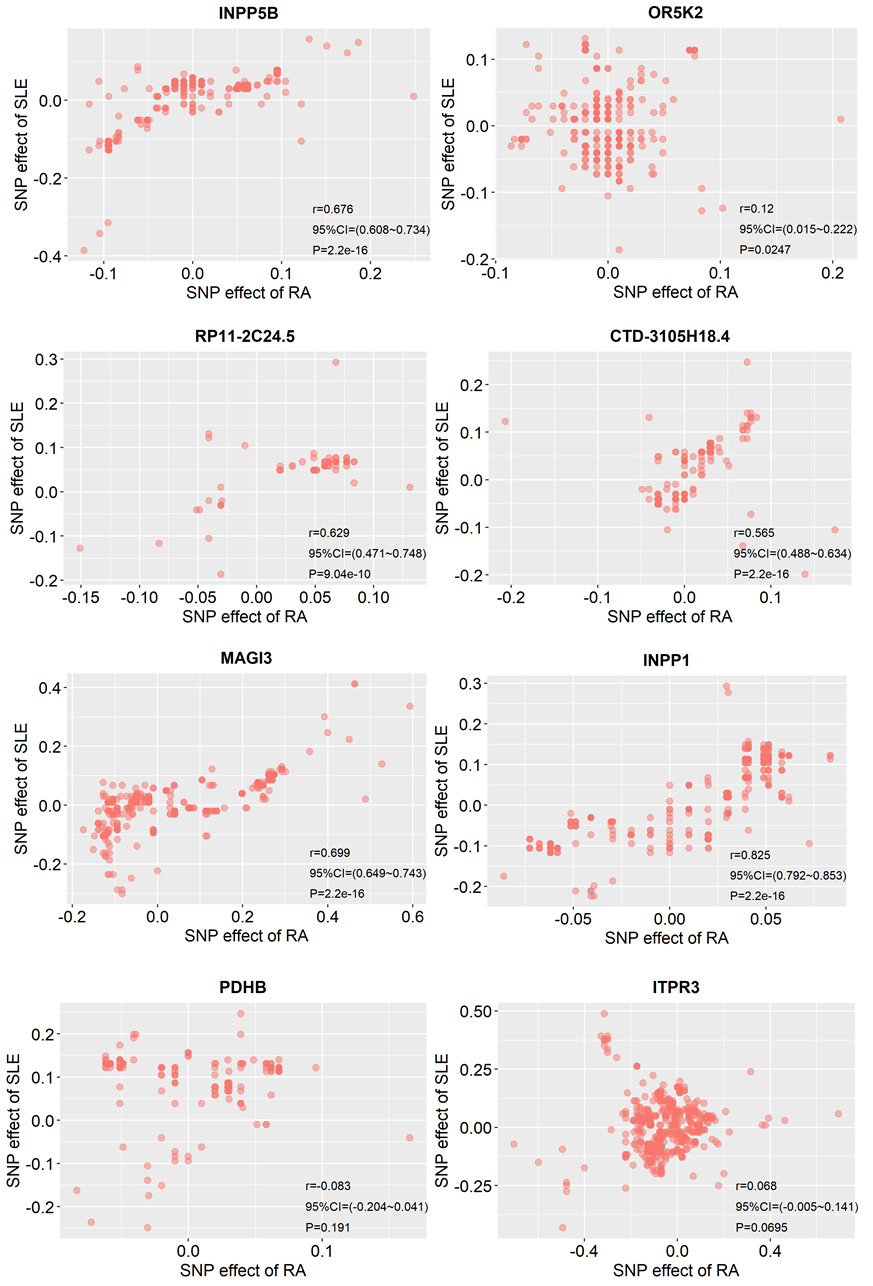
**


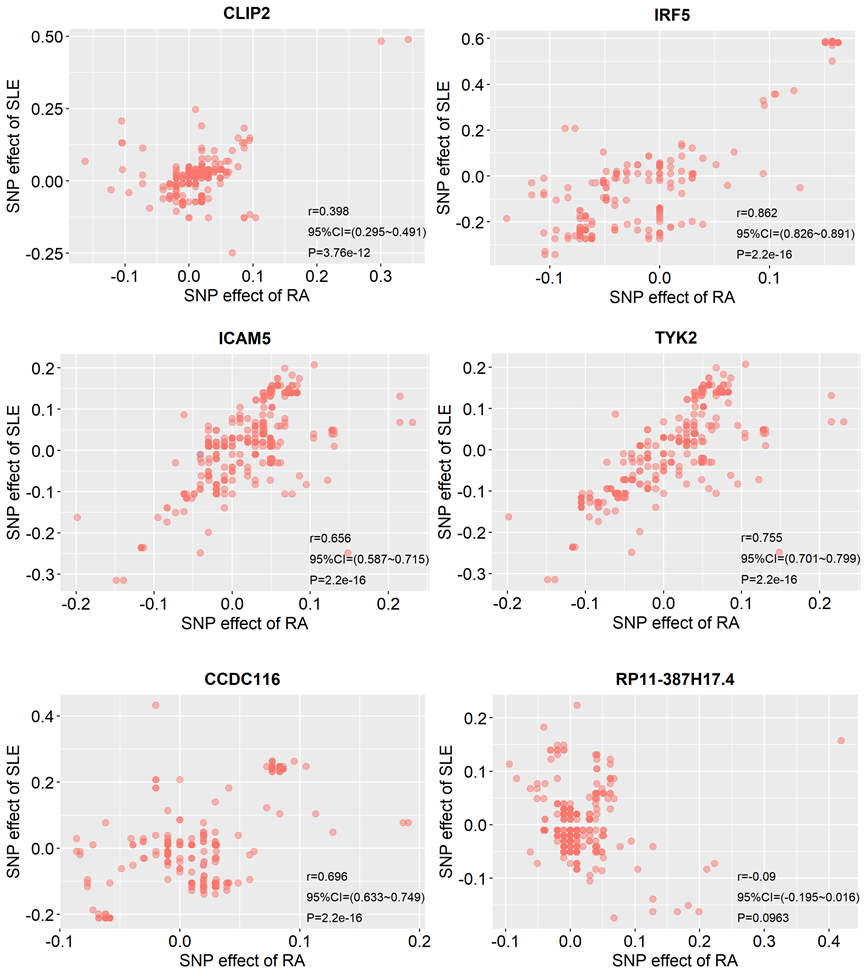


**Supplementary Figure 2.** Correlation of SNP effect sizes of each pleiotropic gene for RA and SLE. For each gene, genetic variants located within either 100 kb upstream of the transcription start site or 100 kb downstream of the transcription end site were extracted and considered as its cis-SNPs.


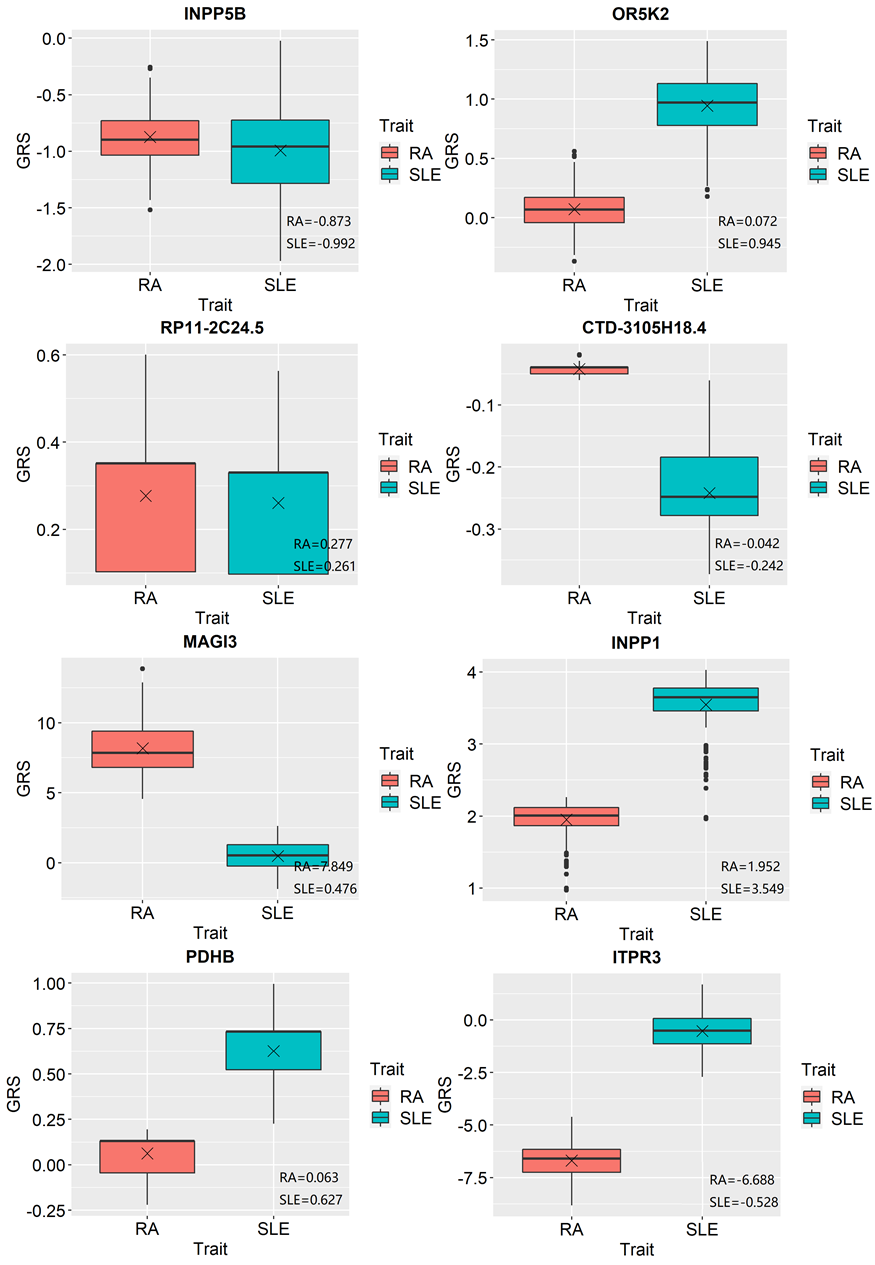

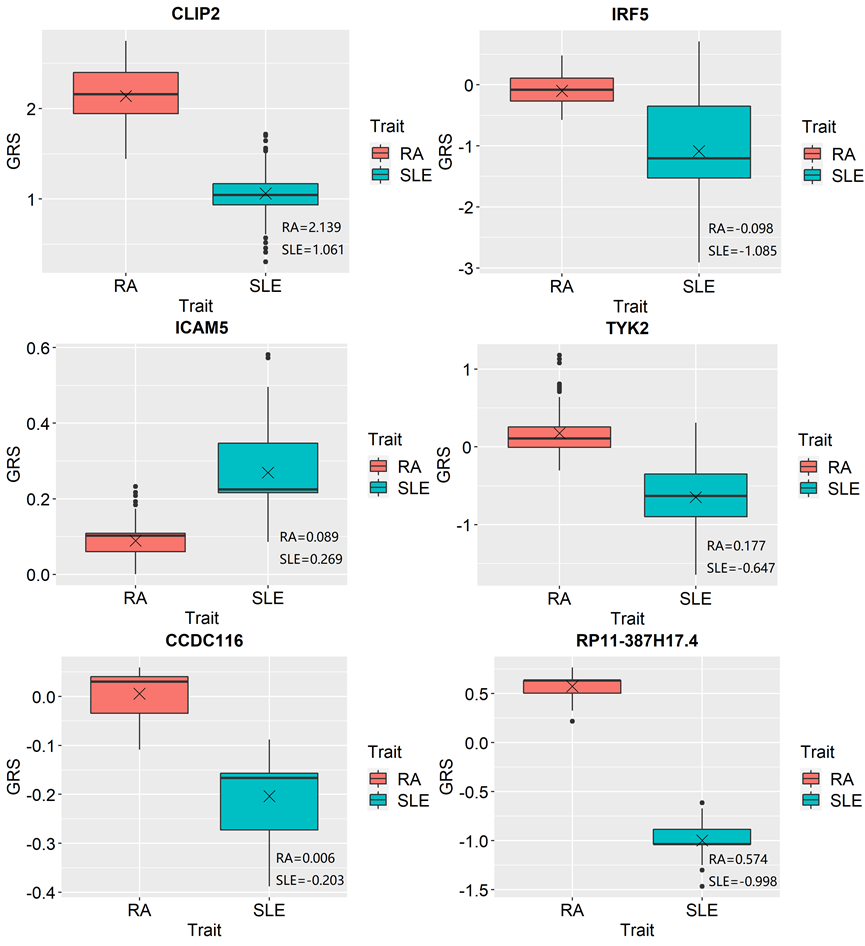


**Supplementary Figure 3.** Boxplot of the genetic risk score of each pleiotropic gene for RA and SLE. For each gene, genetic variants located within either the transcription start site or the transcription end site were extracted and considered as its cis-SNPs. The genetic risk score for pleiotropic gene is calculated as the product of SNP effect sizes and genotypes of 503 European individuals available from the 1000 Genomes Project phase III. The average of the genetic risk score for each disease across individuals is shown in each panel.
